# Bayesian Reinforcement Learning With Limited Cognitive Load

**DOI:** 10.1162/opmi_a_00132

**Published:** 2024-04-03

**Authors:** Dilip Arumugam, Mark K. Ho, Noah D. Goodman, Benjamin Van Roy

**Affiliations:** Department of Computer Science, Stanford University; Center for Data Science, New York University; Department of Psychology, Stanford University; Department of Electrical Engineering, Stanford University; Department of Management Science & Engineering, Stanford University

**Keywords:** Bayesian decision making, efficient exploration, reinforcement learning, multi-armed bandits, information theory, rate-distortion theory

## Abstract

All biological and artificial agents must act given limits on their ability to acquire and process information. As such, a general theory of adaptive behavior should be able to account for the complex interactions between an agent’s learning history, decisions, and capacity constraints. Recent work in computer science has begun to clarify the principles that shape these dynamics by bridging ideas from *reinforcement learning*, *Bayesian decision-making*, and *rate-distortion theory*. This body of work provides an account of *capacity-limited Bayesian reinforcement learning*, a unifying normative framework for modeling the effect of processing constraints on learning and action selection. Here, we provide an accessible review of recent algorithms and theoretical results in this setting, paying special attention to how these ideas can be applied to studying questions in the cognitive and behavioral sciences.

## INTRODUCTION

Cognitive science aims to identify the principles and mechanisms that underlie adaptive behavior. An important part of this endeavor is the development of *normative* theories that specify the computational goals and constraints of an intelligent system (Anderson, 1990; Gershman et al., 2015; Griffiths et al., 2015; Lewis et al., 2014; Marr, 1982). For example, accounts of learning, cognition, and decision-making often posit a function that an organism is optimizing—*e.g.*, maximizing long-term reward or minimizing prediction error—and test plausible algorithms that achieve this—*e.g.*, a particular learning rule or inference process. Historically, normative theories in cognitive science have been developed in tandem with new formal approaches in computer science and statistics. This partnership has been fruitful even given differences in scientific goals (*e.g.*, engineering artificial intelligence versus *reverse*-engineering biological intelligence). Normative theories play a key role in facilitating cross-talk between different disciplines by providing a shared set of mathematical, analytical, and conceptual tools for describing computational problems and how to solve them (Ho & Griffiths, 2022).

**Figure 1.**
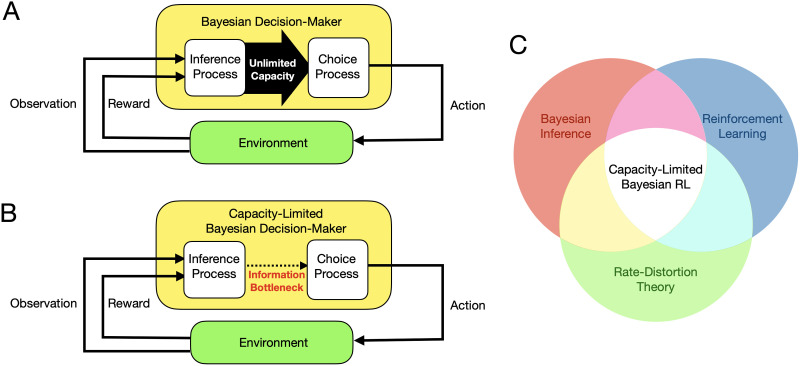
(A) Bayesian learning and decision-making is typically modularized into distinct stages of *inference* and *choice*. That is, the decision-maker is conceptualized as mapping experiences to probabilistic beliefs about the environment (an inference process) and then performing computations based on the resulting beliefs to produce distributions over actions (a choice process). Inference and choice processes are usually specified independently and assume that the channel from one to the other has unlimited capacity (thick solid arrow). (B) In *capacity-limited Bayesian decision-making*, there exists an information bottleneck between inferences and choices (narrow dotted arrow). Given the results of a fixed inference process (*e.g.*, exact or approximate Bayesian inference), the optimal choice process trades off expected rewards and the mutual information (the *rate*) between beliefs about the environment and the distribution over desirable actions. (C) Capacity-limited Bayesian reinforcement learning integrates ideas from *Bayesian inference* (Jaynes, 2003), *reinforcement learning* (Kaelbling et al., 1996), and *rate-distortion theory* (Cover & Thomas, 2012).

This paper is written in the spirit of such cross-disciplinary fertilization. Here, we review recent work in computer science (Arumugam & Van Roy, 2021a, 2022) that develops a novel approach for unifying three distinct mathematical frameworks that will be familiar to many cognitive scientists (Figure 1). The first is *Bayesian inference*, which has been used to study a variety of perceptual and higher-order cognitive processes such as categorization, causal reasoning, and social reasoning in terms of inference over probabilistic representations (Baker et al., 2009; Battaglia et al., 2013; Collins & Frank, 2013; Tenenbaum et al., 2011; Yuille & Kersten, 2006). The second is *reinforcement learning* (Sutton & Barto, 1998), which has been used to model key phenomena in learning and decision-making including habitual versus goal-directed choice as well as trade-offs between exploring and exploiting (Daw et al., 2011; Dayan & Niv, 2008; Radulescu et al., 2019; Wilson et al., 2014). The third is *rate-distortion theory* (Berger, 1971; Shannon, 1959), a subfield of information theory (Cover & Thomas, 2012; Shannon, 1948), which in recent years has been used to model the influence of capacity-limitations in perceptual and choice processes (Lai & Gershman, 2021; Sims, 2016; Zaslavsky et al., 2021; Zénon et al., 2019). All three of these formalisms have been used as normative frameworks in the sense discussed above: They provide general design principles (*e.g.*, rational inference, reward-maximization, efficient coding) that explain the function of observed behavior and constrain the investigation of underlying mechanisms.

Although these formalisms have been applied to analyzing individual psychological processes, less work has used them to study learning, decision-making, and capacity limitations holistically. One reason is the lack of principled modeling tools that comprehensively integrate these multiple normative considerations. The framework of *capacity-limited Bayesian reinforcement learning*, originally developed by Arumugam and Van Roy (2021a, 2022) in the context of machine learning, directly addresses the question of how to combine these perspectives. As its name suggests, the cornerstone of this framework is classic reinforcement learning, which traditionally focuses on idealized decision-making agents determined to synthesize optimal behavior without regard for resource constraints that may adversely impact the efficiency of learning. While the intersection of Bayesian inference and reinforcement learning has also been well-studied in the machine-learning literature (Bellman & Kalaba, 1959; Duff, 2002; Ghavamzadeh et al., 2015) and offers a powerful mechanism for gracefully tackling exploration (Agrawal & Jia, 2017; Osband et al., 2013; Osband & Van Roy, 2017; Strens, 2000), it too only offers consideration for optimal decision-making without regard for agent limitations that may leave optimal behavior highly challenging to obtain or even categorically unachievable. In contrast, while the intersection of rate-distortion theory and reinforcement learning (Abel et al., 2019; Lai & Gershman, 2021; Polani, 2009, 2011; Rubin et al., 2012; Still & Precup, 2012; Tishby & Polani, 2011) does offer one notion of capacity-sensitive behavior, it only specifies an alternative outcome to the traditional optimal policy but fails to prescribe a mechanism for orienting exploration around such a behavior. Consequently, these algorithms only offer insight into the end products of learning but do not clarify how agent limitations impact the dynamics of the learning process itself. By operating at the intersection of these three areas (Figure 1), capacity-limited Bayesian reinforcement learning highlights how capacity constraints impact an agent’s exploration strategy, thereby not only leading to tractable learning outcomes but also influencing the full dynamics of learning over time. Our goal is to review this work and present its key developments in a way that will be accessible to the broader research community and can pave the way for future cross-disciplinary investigations.

Notably, while the capacity constraints accommodated by the work presented in this paper can be quite versatile, a key motivation of this framework is offering a treatment of decision-making subject to constraints on time. Indeed, people often find themselves forced to select from considerably-large action spaces with significantly less time than what is needed to adequately explore all available decisions. When the disparity between total time allotted for learning and total number of actions available becomes sufficiently large, identifying an optimal action becomes entirely infeasible as a learning objective. While one could nevertheless deploy a classic decision-making algorithm in such a setting, acknowledging that it will not succeed in reaching optimal performance, such agents are designed with one of many strategies to address the explore-exploit trade-off. Unfortunately, this exploration mechanism is likely tailored for uncovering information salient to (unachievable) optimal behavior and is not guaranteed to be effective for gathering information about any other alternative, feasible behavior. In contrast, capacity-limited Bayesian decision-making offers a mechanism by which an agent may align exploratory decisions to a feasible behavior under the time constraints at hand.

We present the framework in two parts. First, we discuss a formalization of capacity-limited Bayesian decision-making, beginning with a few simple key tenets that underlie the coupling of Bayesian inference, information theory, and decision making. These core principles come together and allow for the introduction of an information bottleneck between an agent’s beliefs about the world and what it aspires to learn from its interactions with the world. To the extent that exploration is a challenge of information acquisition, this bottleneck serves as a targeting mechanism through which a bounded agent can prioritize which pieces of information to seek out. This motivates a novel family of algorithms for consuming environmental beliefs and an information-constrained target to select actions in a manner that optimally trades off between reward and information. Second, through a series of simple toy simulations, we analyze a specific algorithm: a variant of Thompson Sampling (Thompson, 1933) modified to incorporate such an information bottleneck. Afterwards, we turn more fully to capacity-limited Bayesian reinforcement learning, in which a decision-maker is continuously interacting with and adapting to their environment. We report a mixture of both novel as well as previously-established simulations and theoretical results in several learning settings, including multi-armed bandits as well as continual and episodic reinforcement learning. One feature of this framework is that it provides tools for analyzing how the interaction between capacity-limitations and learning dynamics can influence learning outcomes; in the discussion, we explore how such analyses and our framework can be applied to questions in cognitive science. We also discuss similarities and differences between capacity-limited Bayesian reinforcement learning and existing proposals including information-theoretic bounded rationality (Gottwald & Braun, 2019; Ortega & Braun, 2011), policy compression (Lai & Gershman, 2021), and resource-rational models based on principles separate from information theory (Callaway et al., 2022; Ho et al., 2022; Lieder et al., 2014).

## CAPACITY-LIMITED BAYESIAN DECISION-MAKING

This section provides a preliminary account of capacity-limited Bayesian decision-making. As previously discussed, the incorporation of capacity limitations will be realized through rate-distortion theory; accordingly, we organize the section to separately introduce the elements of distortion and rate before turning our attention to the tension between them that a bounded decision-making agent is expected to negotiate. We conclude the section with a discussion and analysis of a practical algorithm for computing capacity-limited Bayesian decision procedures based on Thompson Sampling.

### Bayesian Inference & Utility

Bayesian or probabilistic models have been used to characterize a range of psychological phenomena, including perception, categorization, feature learning, causal reasoning, social interaction, and motor control (Goodman & Frank, 2016; Itti & Baldi, 2009; Körding & Wolpert, 2004; Ma, 2012). One distinguishing feature of Bayesian models is that they separate learning and decision-making into two stages: *inferring* a function or statistic of the environment and *choosing* an action based on those inferences (Figure 1A). This separation of inference and choice into an independent Bayesian estimator and decision-rule is commonly assumed throughout psychology, economics, and computer science (Kaelbling et al., 1998; Ma, 2019; von Neumann & Morgenstern, 1944). However, even if inference about the environment is exact, exploring to learn good decisions incurs some non-trivial degree of cognitive load and the associated cost or limit on how much those inferences can inform what an agent learns remains unaccounted for. We now turn to extending (Arumugam & Van Roy, 2021a, 2022) the standard Bayesian framework to incorporate such capacity limitations (Figure 1B). Our focus begins purely on the inference process while later (see Thompson Sampling: Combining Bayesian Inference and Decision-Making section) clarifying how these capacity limitations during inference manifest in the choice process of an agent.

The starting point for inference is formalized in terms of an *environment-estimator*, a probability distribution over the unknown environment 𝓔 that is updated based on the experiences of the agent. Formally, given a history of experiences *H*_*t*_ up to time *t*, an environment-estimator *η*_*t*_ is updated according to Bayes’ rule:

$$\eta_{t}\left(𝓔\right)=\mathbb{P}\left(𝓔|H_{t}\right)\propto \mathbb{P}\left(H_{t}|𝓔\right)\mathbb{P}\left(𝓔\right),$$ (1)

where ℙ(*H*_*t*_ | 𝓔) is the likelihood of history *H*_*t*_ under 𝓔 and ℙ(𝓔) is the prior probability assigned to 𝓔.

While the environment 𝓔 denotes the cumulative knowledge an agent maintains about the world, the goal or objective an agent aspires to learn about through its interactions within the environment is formalized as a *learning target χ*. That is, if 𝓔 denotes the information an agent retains, then *χ* denotes the information an agent seeks out through its interactions (Lu et al., 2023). This target is a (potentially stochastic) function of the unknown environment that can be represented as a conditional probability distribution over actions, given the identity of the environment, *δ*(*χ* | 𝓔) = ℙ(*χ* | 𝓔). Intuitively, for a particular realization of the environment 𝓔 = *θ*, the learning target *χ* ∼ *δ*(· | 𝓔) = *θ* characterizes the agent’s beliefs about what it should learn when treating environment *θ* as reality.

Suppose we have a real-valued utility function *U*(*a*, *θ*) that quantifies the performance or goodness of an action *a* ∈ 𝒜 for a particular realization of the environment 𝓔 = *θ* (later we discuss reinforcement learning and will consider specific utility functions that represent reward and/or value). A standard and widely-studied choice of learning target is an optimal action *A*^⋆^ ∈ $\underset{a\in 𝒜}{\arg\;\max}$ *U*(*a*, 𝓔) that maximizes utility. For an unconstrained agent with unlimited capacity, there is perhaps no reason to entertain any other learning target besides *A*^⋆^. In the next section, however, we use information theory to articulate the associated cost of exploring to learn an optimal decision *A*^⋆^, which may be infeasible for a capacity-limited decision-making agent.

### The Duality Between Uncertainty & Information

While the previous section establishes the desirability of a learning target within some environment through its utility, this section provides a parallel account for the cost of learning through information. As a simple example, suppose an agent wishes to learn about the outcome of a coin flip *χ* ∼ Bernoulli(𝓔) from a coin with unknown bias 𝓔 ∈ [0, 1]. Note that a trick coin with 𝓔 = 1 would result in a target *χ* = *f*(𝓔) = HEADS that is just a deterministic function *f* always returning HEADS. On the other hand, for a fair coin 𝓔 = 0.5, the target is now a random function *χ* = *g*(𝓔) = $\left\{\begin{array}{ll} \mathrm{HEADS} & \text{with}\;\text{probability}\;0.5\\ \mathrm{TAILS} & \text{with}\;\text{probability}\;0.5 \end{array}\right.$. The cumulative randomness present in *χ* stems not only from possibly being a non-deterministic function but also from its dependence on 𝓔, which is itself a random variable.

We now turn our attention to the role of information theory (Cover & Thomas, 2012; Shannon, 1948), giving verbal descriptions of the salient quantities and deferring precise mathematical definitions to the appendix (please see Appendix A). The *entropy* ℍ(*χ*) of *χ* quantifies all uncertainty in the agent’s mind about the outcome of the coin flip. Equivalently, an agent that obtains these ℍ(*χ*) bits of information would have zero uncertainty and identify the flip outcome exactly. However, even if the agent had perfect knowledge of the environment 𝓔 to distinguish between a biased or fair coin, there could still be residual uncertainty left over simply because the coin flip is an inherently random outcome (such as in the fair coin scenario above). We can quantify uncertainty with the provision of such knowledge through conditioning and examine the *conditional entropy* of the flip outcome given the coin bias ℍ(*χ* | 𝓔). In general, if the learning target happens to be a deterministic function of the environment (*χ* = *f*(𝓔), for deterministic *f*), then a well-known fact of information theory already establishes that ℍ(*χ* | 𝓔) = 0. If not, however, then ℍ(*χ* | 𝓔) ≥ 0 and, due to the conditioning, this residual uncertainty cannot be eliminated by making decisions and collecting more interaction data from the environment 𝓔. Consequently, while the entropy ℍ(*χ*) quantifies all of the agent’s uncertainty in the learning target, the conditional entropy ℍ(*χ* | 𝓔) captures only the irreducible or aleatoric uncertainty (Der Kiureghian & Ditlevsen, 2009) the agent has in *χ* due to random noise.

It would be somewhat illogical for a decision-making agent, in the course of trying to resolve its own uncertain beliefs about the coin flip, to factor in the irreducible uncertainty that will always be present in a possibly stochastic outcome. Fortunately, the *mutual information* between the environment and target 𝕀(𝓔; *χ*) emerges as a mechanism for quantifying the agent’s reducible or epistemic uncertainty present in its internal beliefs about the learning target *χ* due to its own lack of knowledge, rather than sheer randomness:

$$\underset{\text{Epistemic}}{\underbrace{𝕀\left(𝓔;\chi \right)}}=\underset{\text{Total}}{\underbrace{ℍ\left(\chi \right)}}-\underset{\text{Aleatoric}}{\underbrace{ℍ\left(\chi |𝓔\right)}}.$$

From this, we see that mutual information quantifies all of the “usable” information about a target *χ* available for an agent to learn through its interactions with the environment 𝓔. When the agent no longer has any epistemic uncertainty in *χ*, this is akin to saying that its beliefs about *χ* have converged to the true value and the environment 𝓔 has no more usable information to offer an agent learning about the target, 𝕀(𝓔; *χ*) = 0; thus, in essence, the agent has finished learning *χ* to completion. In the vernacular of information theory, a learning target *χ* is characterized by its associate conditional probability distribution or channel *δ* and the mutual information or rate of this channel quantifies the number of bits transmitted or communicated on average. The notion of rate comes from rate-distortion theory, a sub-field of information theory that studies how to design efficient but lossy coding schemes (Berger, 1971; Shannon, 1959). In our context, this gives a precise mathematical form for how much residual uncertainty in a target (the channel output) remains within the environment (the channel input). In the context of this paper, a central assumption of this framework is that a learning target attributed to a higher rate is more cognitively costly.

The exploration strategy employed by a decision-making agent is responsible for the acquisition of these 𝕀(𝓔; *χ*) bits of information over the course of learning. Thus, intuitively, it follows that some targets are easier to learn than others. More concretely, for two targets *χ*_1_ and *χ*_2_, having 𝕀(𝓔; *χ*_1_) ≤ 𝕀(𝓔; *χ*_2_) implies that an agent is closer to resolving its uncertainty in target *χ*_1_ than *χ*_2_, thereby implying *χ*_1_ is easier to learn. Of course, if *χ*_2_ allows an agent to obtain significantly higher utility relative to what is possible with the knowledge encoded in *χ*_1_, then perhaps it is worthwhile for a limited agent to pursue the more challenging target *χ*_2_. The next section discusses how such an agent can negotiate this tension between information and utility to reduce cognitive load when deciding what to learn.

### Balancing Between Bits & Utility

Under ideal conditions, decision-making agents pursue optimal behavior to maximize utility without regard for the difficulty of learning. Unlimited capacity and resources implies that acquiring the 𝕀(𝓔; *A*^⋆^) bits needed to identify an optimal decision is always feasible. In contrast, a capacity-limited decision-making agent may likely find the same exploration problem for *A*^⋆^ too onerous and must instead be willing to sacrifice some amount of utility in exchange for a more tractable learning target. Given current beliefs about the environment 𝓔, a bounded agent might engage with the following constrained optimization problem to balance between these tensions

$$𝒟\left(R\right)=\max_{\chi }𝔼\left[U\left(\chi ,𝓔\right)\right]\;\text{such}\;\text{that}\;𝕀\left(𝓔;\chi \right)\leq R,$$

for some capacity limit *R* ∈ ℝ_≥0_. For a fixed capacity *R*, the solution 𝒟(*R*) to this optimization problem characterizes a fundamental limit on the maximum utility realizable by any decision-making agent that can only hope to learn exactly *R* bits of information from the environment.

Practical models for such capacity-limited agents may find it useful to modify the above problem in two ways. First, by recognizing that maximizing over all possible learning targets *χ* is equivalent to maximizing over conditional probability distributions *δ*(*χ* | 𝓔). Second, rather than dealing in the constrained optimization problem, solving the unconstrained optimization problem

$$\max_{\delta \left(\chi |𝓔\right)}𝔼\left[U\left(\chi ,𝓔\right)\right]-\lambda 𝕀\left(𝓔;\chi \right),$$

where *λ* ∈ ℝ_≥0_ is now a hyperparameter used to communicate a desired trade-off between utility and capacity. As *λ* ↓ 0, an agent falls back to capacity-insensitive behavior and prioritizes performance, drawing closer and closer to identifying an optimal action *A*^⋆^. Alternatively, as *λ* ↑ ∞, an agent pursues increasing simpler targets that demand exploring for fewer bits of information from the environment at the cost of worsening utility, eventually recovering the uniform random action $\bar{A}$ such that *δ*($\bar{A}$ = *a* | 𝓔) = $\frac{1}{|𝒜|}$, for all *a* ∈ 𝒜; due to the non-negativity of mutual information (𝕀(𝓔; *χ*) ≥ 0, for all *χ*), it follows that an agent behaving by sampling actions uniformly at random is the easiest to learn as 𝕀(𝓔; $\bar{A}$) = 0. Of course, under the lens of the earlier section, an agent that aspires to achieve uniform random action selection is unlikely to derive much utility from such behavior. On the other hand, a capacity-limited learner may struggle to explore and acquire all salient bits of information needed to be optimal 𝕀(𝓔; *A*^⋆^).

For the ease of exposition, let $\tilde{A}$ denote the learning target between *A*^⋆^ and $\bar{A}$ achieved by solving the above optimization problem for an arbitrary choice of *λ*. How quickly a decision-making agent can obtain these 𝕀(𝓔; $\tilde{A}$) bits of information over time will ultimately determine the speed of learning. Recall from the previous section that, at any time period *t* with history *H*_*t*_, having zero epistemic uncertainty given the random history *H*_*t*_, 𝕀_*t*_(𝓔; $\tilde{A}$) = 0, implies the completion of learning $\tilde{A}$. Thus, one could define the sample complexity of learning $\tilde{A}$ within a total *T* ∈ ℕ time periods as

$$𝔼\left[\sum_{t=1}^{T}𝟙\left({𝕀}_{t}\left(𝓔;\tilde{A}\right)>0\right)\right],$$

where 𝟙(·) is the binary indicator that returns 1 if the input proposition is true and 0 otherwise. At each time period *t*, this quantity examines how much lingering epistemic uncertainty an agent has in the target $\tilde{A}$ despite its interaction history *H*_*t*_ with the environment thus far, 𝕀_*t*_(𝓔; $\tilde{A}$). As time is, ultimately, the scarce resource a capacity-limited Bayesian decision-making agent must negotiate, *λ* emerges as a knob for tailoring $\tilde{A}$ to respect this constraint. If *λ* is chosen large enough such that $\tilde{A}$ = $\bar{A}$, then an agent will find an associated sample complexity of zero across all time periods and irrespective of its own action selection; this yields the rather unimpressive conclusion that learning how to select actions uniformly at random requires no interaction data despite being tremendously sub-optimal. At the other end of the spectrum, having *λ* = 0 and $\tilde{A}$ = *A*^⋆^ requires a combination of sufficiently many time periods *T* to learn as well as prudent exploration to resolve all epistemic uncertainty in optimal behavior and obtain low sample complexity. As an agent increases *λ*, $\tilde{A}$ moves along this optimal complexity-utility trade-off resulting in a broad spectrum of near-optimal behavior incurring smaller sample complexity as sub-optimality increases. Of course, regardless of where a capacity-limited agent ends up, one question that remains is how the resulting target $\tilde{A}$ should impact action selection?

### Thompson Sampling: Combining Bayesian Inference and Decision-Making

Unlike classic information theory applications in compression and communication where all bits are created equal to be transmitted with identical priority, decision makers take actions to learn about a particular target *χ* and not all information about the world revealed by a decision is guaranteed to provide target-relevant information. Prudent strategies for exploration tailored for a particular *χ* capitalize on the agent’s current beliefs about the world 𝓔 given the history of interaction thus far *H*_*t*_ to select actions that either succeed in revealing target-relevant information or, when such information has been exhausted from the environment, 𝕀_*t*_(*χ*; 𝓔) = 0, allow the agent to exploit what it has learned. In this section, we review an algorithm known as Thompson Sampling for establishing a powerful link between the agent’s inference process that maintains beliefs about the world 𝓔 coupled with a learning target *χ* to direct the choice process.

Recall from the previous section that we let $\tilde{A}$ denote a learning target *χ* chosen to achieve an optimal trade-off between complexity and utility. At this point, all that remains is to prescribe a mechanism by which an agent can turn beliefs about the environment 𝓔 and a desired learning target $\tilde{A}$ into an action choice *A*_*t*_ ∈ 𝒜 that is ultimately executed in the true environment. This requires specification of a policy *π* that examines the history *H*_*t*_ and prescribes a distribution over actions from which *A*_*t*_ can be sampled: *A*_*t*_ ∼ *π*(· | *H*_*t*_). While there are many options for how to derive such a policy using current beliefs about the world and a target, Thompson Sampling (Russo & Van Roy, 2016; Thompson, 1933) is a simple, provably-efficient, and widely-deployed choice for handling exploration. Thompson Sampling proceeds via the probability-matching principle whereby an agent only executes actions according to the probability that they are desirable target actions. Formally, this means that

$$\pi \left(a|H_{t}\right)=\mathbb{P}\left(A_{t}=a|H_{t}\right)=\mathbb{P}\left(\chi =a|H_{t}\right),\;\forall a\in 𝒜.$$

An unbounded agent free from the burdens of capacity limitations always acts in pursuit of an optimal action *χ* = *A*^⋆^ and, indeed, this special case of the probability-matching principle shown above has been widely studied in the literature (Agrawal & Goyal, 2012, 2013; Russo & Van Roy, 2016). Observe that the moment an agent’s beliefs about the world have been sufficiently informed to determine that some action *a* ∈ 𝒜 cannot be optimal, Thompson Sampling immediately reduces the probability of taking such a sub-optimal action to zero ℙ(*A*_*t*_ = *a* | *H*_*t*_) = ℙ(*A*^⋆^ = *a* | *H*_*t*_) = 0.

While the formal theoretical proof of a Thompson Sampling agent’s efficacy in handling exploration is comforting (Russo & Van Roy, 2016), part and parcel to its widespread practical use (Chapelle & Li, 2011) is the computational efficiency of its implementation. Specifically, by marginalizing over the environment 𝓔, we have

$$\pi \left(A_{t}|H_{t}\right)=\mathbb{P}\left(\chi |H_{t}\right)={𝔼}_{\theta ∼\eta_{t}}\left[\delta \left(\chi |𝓔=\theta \right)\eta_{t}\left(𝓔=\theta \right)\right].$$

Thus, to implement Thompson Sampling as shown in Algorithm 1, an agent need only draw one plausible hypothesis about 𝓔 from its internal beliefs *θ* ∼ *η*_*t*_ (formally, a *n* = 1 single-sample, Monte-Carlo approximation of the above expectation) followed by sampling a target action *A*_*t*_ ∼ *δ*(· | 𝓔) = *θ* conditioned on the environment sample. Once again, the literature typically restricts focus to optimal actions *χ* = *A*^⋆^ by assumption such that Thompson Sampling can be interpreted as simply drawing one hypothesis about the true world and acting optimally with respect to this sample. More broadly, Thompson Sampling provides a strong coupling between how an agent explores the environment and what the agent aims to learn through those interactions.


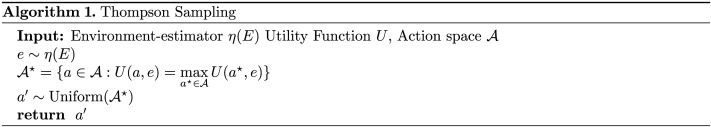


Of course, other more-elaborate possibilities do exist in the literature (Russo & Van Roy, 2014, 2018a), however this paper focuses in on Thompson Sampling as a simple yet effective choice among them. Different decision-rules are distinguished by the type of representation they use and the algorithms that operate over those representations. For example, some decision-rules only use a *point-estimate* of each action’s expected reward, such as *reward maximization*, *ε*-*greedy reward maximization* (Cesa-Bianchi & Fischer, 1998; Kuleshov & Precup, 2014; Vermorel & Mohri, 2005), *Boltzmann*/*softmax* action selection (Asadi & Littman, 2017; Kuleshov & Precup, 2014; Littman, 1996), or *upper-confidence bound* (UCB) action selection (Auer, 2002; Auer et al., 2002; Kocsis & Szepesvári, 2006). Some of these rules also provide parameterized levels of “noisiness” that facilitate random exploration—*e.g.*, the probability of selecting an action at random in *ε*-greedy, the temperature in a Boltzmann distribution, and the bias factor in UCB. In the Bayesian setting, decision-rules like Thompson Sampling can take advantage of epistemic uncertainty to guide exploration. Additionally, humans often display key signatures of selecting actions via Thompson Sampling (Gershman, 2018; Vulkan, 2000; Wozny et al., 2010). In short, classic Thompson Sampling is a simple, robust, and well-studied Bayesian algorithm that is, by design, tailored to an optimal learning target *A*^⋆^; this, however, assumes that a decision-making agent has the unlimited capacity needed to acquire all bits of information relevant to *A*^⋆^, 𝕀(𝓔; *A*^⋆^).

One instantiation of a capacity-limited Bayesian decision-making agent combines rate-distortion theory and Thompson Sampling by first computing a learning target $\tilde{A}$ that optimally strikes some balance between complexity and utility before choosing an action via probability matching with respect to this target. Such an agent employs Blahut-Arimoto Satisficing Thompson Sampling (BLASTS), an algorithm first proposed by Arumugam and Van Roy (2021a). In order to approximate an optimal decision-rule given current beliefs about the world 𝓔 and rate parameter *λ* ≥ 0, BLASTS (whose pseudocode appears as Algorithm 2) performs three high-level procedures. First, it approximates the environment distribution by drawing *Z* ∈ ℕ Monte-Carlo samples from *η* and proceeding with this discrete empirical distribution. Second, it uses Blahut-Arimoto—a classic algorithm from the rate-distortion theory literature (Arimoto, 1972; Blahut, 1972) based on convex optimization (Boyd & Vandenberghe, 2004)—to iteratively compute the (globally) optimal learning target $\tilde{A}$. Finally, it uniformly samples one of the *Z* initially drawn environment configurations *e*′ and then samples an action *a*′ from the computed decision-rule conditioned on that realization *e*′ of the environment.


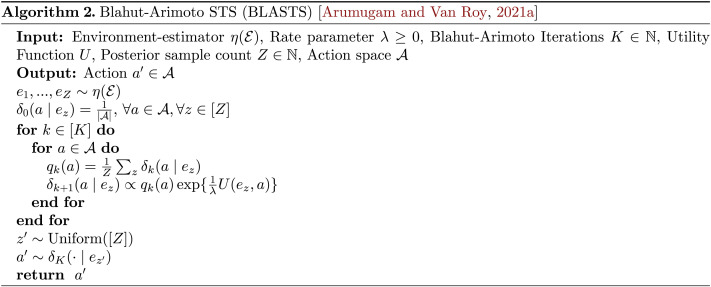


**Figure 2.**
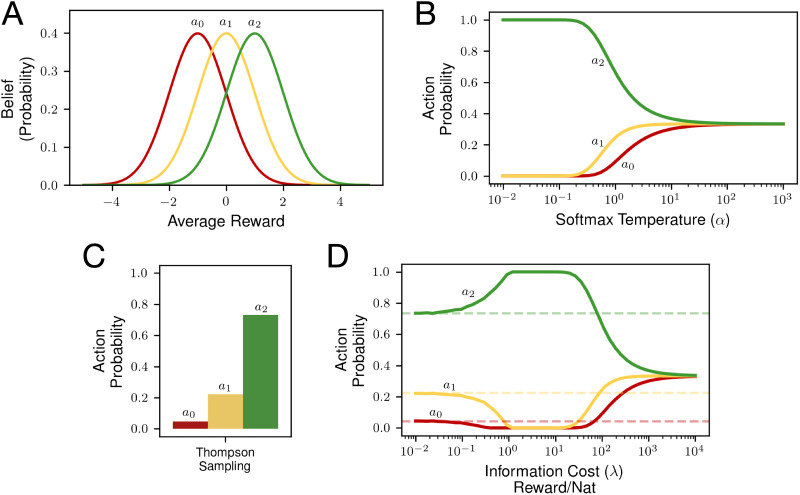
Capacity-limited decision-making in a three-armed bandit. (A) Bayesian decision-makers represent probabilistic uncertainty over their environment. Shown are Gaussian beliefs for average rewards for three actions, *a*_0_, *a*_1_, and *a*_2_, with location parameters *μ*_0_ = − 1, *μ*_1_ = 0, *μ*_2_ = 1, and standard deviations *σ*_*i*_ = 1 for *i* = 0, 1, 2. (B) A non-Bayesian decision-rule is the Boltzmann or soft-max distribution (Littman, 1996), which has a temperature parameter *α* > 0. For the values in panel A, as *α* → 0, the action with the highest expected reward is chosen more deterministically; as *α* → ∞, actions are chosen uniformly at random. The Boltzmann decision-rule ignores distributional information. (C) An alternative decision-rule that is sensitive to distributional information is Thompson Sampling (Thompson, 1933), which implements a form of *probability matching* that is useful for exploration (Russo & Van Roy, 2016). Shown are the Thompson Sampling probabilities based on *N* = 10,000 samples. Thompson Sampling has no parameters. (D) In capacity-limited decision-making, action distributions that are more tightly coupled to beliefs about average rewards—i.e., those with higher mutual information or *rate*—are penalized. The parameter *λ* ≥ 0 controls the penalty and represents the cost of information in rewards per nat. Blahut-Arimoto Satisficing Thompson Sampling (BLASTS) (Arumugam & Van Roy, 2021a) generalizes Thompson Sampling by finding the estimate-to-action channel that optimally trades off rewards and rate for a value of *λ*. In the current example, when 0 < *λ* ≤ 10^−1^, information is cheap and BLASTS implements standard Thompson Sampling; when 10^−1^ ≤ *λ* ≤ 10^1^, BLASTS prioritizes information relevant to maximizing rewards and focuses on exploiting arms with higher expected reward, eventually only focusing on the single best; when *λ* ≥ 10^1^, information is too expensive to even exploit, so BLASTS resembles a Boltzmann distribution with increasing temperature, tending towards a uniform action distribution—that is, one that is completely uninformed by beliefs. Solid lines represent action probabilities according to BLASTS (*Z* = 50,000); dotted lines are standard Thompson Sampling probabilities for reference.

One can observe that a BLASTS agent with no regard for respecting capacity limitations (*λ* = 0) will recover Thompson Sampling as a special case. However, as an agent navigates the space of learning targets to find a suitable balance between *complexity* and *utility* via a setting of *λ*, this generalized version of Thompson Sampling offers one prescription for how this shift in learning target should impact the dynamics of exploration. To illustrate this behavior, we conducted two sets of simulations that manipulated these factors in simple three-armed bandit tasks. Our first set of simulations examined the effect of different values of the rate parameter λ, which intuitively corresponds to the *cost of information* measured in units of utils per nat. We calculated the marginal action distribution, *π*(*a*) = ∑_*e*_ *δ*^⋆^(*a* | *e*)*η*(*e*), where the belief distribution over average rewards for the three arms was represented by three independent Gaussian distributions respectively centered at −1, 0, and 1; all three distributions had a standard deviation of 1 (Figure 2A).

Even on this simple problem, BLASTS displays three qualitatively different regimes of action selection when varying the rate parameter, *λ*, from 10^−2^ to 10^4^. When information is inexpensive (*λ* < 10^−1^), the action distribution mimics the exploratory behavior of Thompson Sampling (consistent with theoretical predictions [Arumugam & Van Roy, 2021a]). As information becomes moderately expensive (10^−1^ ≤ *λ* ≤ 10^1^), BLASTS focuses channel capacity on the actions with higher expected utility by first reducing its selection of the worst action in expectation (*a*_0_) followed by the second-worst/second-best action in expectation (*a*_1_), which results in it purely exploiting the best action in expectation (*a*_2_). Finally, as the util per nat becomes even greater (*λ* ≥ 10^1^) BLASTS produces actions that are *uninformed* by its beliefs about the environment. This occurs in a manner that resembles a Boltzmann distribution with increasing temperature, eventually saturating at a uniform distribution over actions. These patterns are visualized in Figure 2B–D, which compare action probabilities for Boltzmann, Thompson Sampling, and BLASTS.

**Figure 3.**
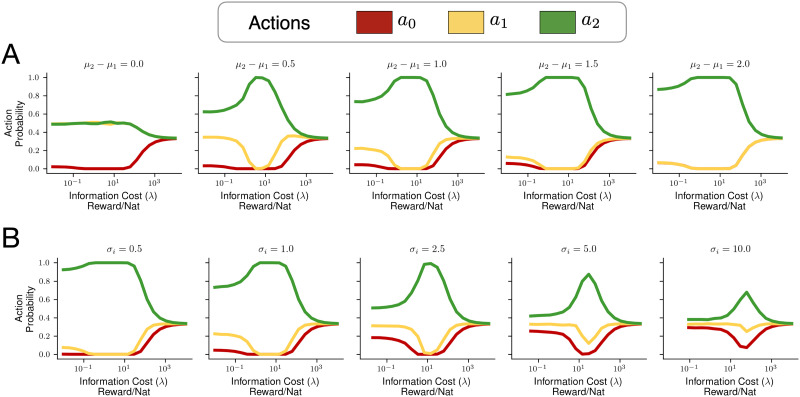
Blahut-Arimoto Satisficing Thompson Sampling (BLASTS) for different beliefs about average rewards in a three-armed bandit. (A) BLASTS is sensitive to the *action gap*—the difference between the expected reward of the highest and second highest actions. Shown are action probability by information cost curves when *μ*_1_ from the example in Figure 2A is set to values in {−1.0, 0.5, 0.0, 0.5, 1.0} and all other belief parameters are held constant. (B) BLASTS is also sensitive to the degree of uncertainty—*e.g.*, the standard deviation of average reward estimates for each action. Shown are action probability / information cost curves when the standard deviation for each arm in Figure 2, *σ*_*i*_, *i* = 0, 1, 2 is set to different values.

Our second set of simulations examine the relationship between the cost of information *λ* and BLASTS action probabilities for different environment-estimates. Specifically, we first examined the effect of changing beliefs about the *action gap*, the difference between the best and second-best action in expectation (Agrawal & Goyal, 2012, 2013; Auer et al., 2002; Bellemare et al., 2016; Farahmand, 2011). As shown in Figure 3A, when the action gap is lower (corresponding to a more difficult decision-making task), BLASTS chooses the optimal action with lower probability for all values of *λ*. In addition, we examined the effect of changing uncertainty in the average rewards by setting different standard deviations for beliefs about the arms. Figure 3B shows that as uncertainty increases, BLASTS is less likely to differentially select an arm even in the “exploitation” regime for moderate values of *λ*. Sensitivity to both the action gap and uncertainty are key features of BLASTS that derive from the fact that it uses distributional information to guide decision-making, unlike decision-rules such as *ε*-greedy or Boltzmann softmax.

Since BLASTS is essentially a parameterized version of Thompson Sampling, it can be used as an alternative decision rule for fitting human data (Wilson & Collins, 2019). Specifically, one approach to using BLASTS would be to jointly fit parameters associated with the inference process (e.g., a participant’s priors about the task) as well as the information cost (*λ*). An important direction for future work will be to validate such an approach and develop efficient algorithms for parameter estimation from participant data.

In the standard formulation of Bayesian decision-making, it is assumed that an agent has unbounded capacity and, therefore, optimal behavior *A*^⋆^ is always achievable. By extending ideas from rate-distortion theory, Arumugam and Van Roy (2021a) defined a notion of capacity limitation applicable to a broader space of learning targets as well as an efficient algorithm for finding such optimal, capacity-limited targets through a variant of Thompson Sampling (BLASTS). In this section, we analyzed how choice distributions change as a function of the cost of information and current environment estimates, which provides some intuition for how capacity-limitations affect choice from the agent’s *subjective* point of view. In the next section, we take a more *objective* point of view by studying the learning dynamics that arise when capacity-limited agents interact with an environment over time.

## CAPACITY-LIMITED BAYESIAN REINFORCEMENT LEARNING

The preceding section provides a cursory overview of how rate-distortion theory accommodates capacity-limited learning within a Bayesian decision-making agent. In this section, we aim to provide mathematically-precise instantiations of the earlier concepts for two distinct problem classes: **(1)** continual or lifelong learning and **(2)** multi-armed bandits; we defer a presentation of our framework applied to episodic Markov decision processes to the appendix. Our aim is to provide a coherent, cohesive narrative for those problem settings that have been examined separately in prior work (Arumugam & Van Roy, 2021a, 2021b, 2022) while also providing a novel extension to the continual learning setting. For the clarity of exposition, a mathematically-inclined reader should consult the appendix for details on notation, definitions of information-theoretic quantities, and all theoretical results.

### Continual Learning

At the most abstract level, we may think of a decision-making agent faced with a continual or lifelong learning setting (Abel et al., 2018; Brunskill & Li, 2013, 2015; Isele et al., 2016; Konidaris & Barto, 2006; Lazaric & Restelli, 2011; Thrun & Schwartz, 1994; Wilson et al., 2007) within a single, stationary environment, which makes no further assumptions about Markovity or episodicity; such a problem formulation aligns with those of Lu et al. (2023) and Dong et al. (2022), spanning multi-armed bandits and reinforcement-learning problems (Lattimore & Szepesvári, 2020; Sutton & Barto, 1998).

#### Problem Formulation.

We adopt a generic agent-environment interface where, at each time period *t*, the agent executes an action *A*_*t*_ ∈ 𝒜 within an environment 𝓔 ∈ *θ* that results in an associated next observation *O*_*t*_ ∈ 𝒪. This sequential interaction between agent and environment yields an associated history^1^ at each timestep *t*, *H*_*t*_ = (*O*_0_, *A*_1_, *O*_1_, …, *A*_*t*−1_, *O*_*t*−1_) ∈ 𝓗, representing the action-observation sequence available to the agent upon making its selection of its current action *A*_*t*_. We may characterize the overall environment as 𝓔 = 〈𝒜, 𝒪, *ρ*〉 ∈ *θ* containing the action set 𝒜, observation set 𝒪, and observation function *ρ* : 𝓗 × 𝒜 → Δ(𝒪), prescribing the distribution over next observations given the current history and action selection: *ρ*(*O*_*t*_ | *H*_*t*_, *A*_*t*_) = ℙ(*O*_*t*_ | 𝓔, *H*_*t*_, *A*_*t*_).

An agent’s policy *π* : 𝓗 → Δ(𝒜) encapsulates the relationship between the history encountered in each timestep *H*_*t*_ and the executed action *A*_*t*_ such that *π*_*t*_(*a*) = ℙ(*A*_*t*_ = *a* | *H*_*t*_) assigns a probability to each action *a* ∈ 𝒜 given the history. Preferences across histories are expressed via a known reward function *r* : 𝓗 × 𝒜 × 𝒪 → ℝ so that an agent enjoys a reward *R*_*t*_ = *r*(*H*_*t*_, *A*_*t*_, *O*_*t*_) on each timestep. Given any finite time horizon *T* ∈ ℕ, the accumulation of rewards provide a notion of return $\sum_{t=1}^{T}$ *r*(*H*_*t*_, *A*_*t*_, *O*_*t*_). To develop preferences over behaviors and to help facilitate action selection, it is often natural to associate with each policy *π* a corresponding expected return or action-value function *Q*^*π*^ : 𝓗 × 𝒜 → ℝ across the horizon *T* as *Q*^*π*^(*h*, *a*) = 𝔼$\left[\sum_{t=1}^{T}r\left(H_{t},A_{t},O_{t}\right)|H_{0}=h,A_{0}=a,𝓔\right]$, where the expectation integrates over the randomness in the policy *π* as well as the observation function *ρ*. Traditionally, focus has centered on agents that strive to achieve the optimal value within the confines of some policy class Π ⊆ {𝓗 → Δ(𝒜)}, *Q*^⋆^(*h*, *a*) = $\underset{\pi \in \Pi }{\sup}$ *Q*^*π*^(*h*, *a*), ∀(*h*, *a*) ∈ 𝓗 × 𝒜. The optimal policy then follows by acting greedily with respect to this optimal value function: *π*^⋆^(*h*) = $\underset{a\in 𝒜}{\arg\;\max}$ *Q*^⋆^(*h*, *a*).

Observe that when rewards and the distribution of the next observation *O*_*t*_ depend only on the current observation-action pair (*O*_*t*−1_, *A*_*t*_), rather than the full history *H*_*t*_, we recover the traditional Markov Decision Process (Bellman, 1957; Puterman, 1994) studied throughout the reinforcement-learning literature (Sutton & Barto, 1998). Alternatively, when these quantities rely solely upon the most recent action *A*_*t*_, we recover the traditional multi-armed bandit (Bubeck & Cesa-Bianchi, 2012; Lai & Robbins, 1985; Lattimore & Szepesvári, 2020). Regardless of precisely which of these two problem settings one encounters, a default presumption throughout both literatures is that an agent should always act in pursuit of learning an optimal policy *π*^⋆^. Bayesian decision-making agents (Bellman & Kalaba, 1959; Duff, 2002; Ghavamzadeh et al., 2015) aim to achieve this by explicitly representing and maintaining the agent’s current knowledge of the environment, recognizing that it is the uncertainty in the underlying environment 𝓔 that drives uncertainty in optimal behavior *π*^⋆^. A Bayesian learner reflects this uncertainty through conditional probabilities *η*_*t*_(*e*) ≜ ℙ(𝓔 = *e* | *H*_*t*_), ∀*e* ∈ Θ aimed at estimating the underlying environment. The problem of explorations centers around how an agent operationalizes its beliefs about the world *η*_*t*_ in order to select actions reveal information salient to good decision-making.

#### Rate-Distortion Theory for Target Actions.

The core insight of this work is recognizing that a delicate balance between the amount of information an agent seeks out through its interactions (*cognitive load*) and the quality of decision-making with that information (*utility*) can be aptly characterized through rate-distortion theory, providing a formal framework for capacity-limited decision making. At each time period *t* ∈ [*T*], the agent’s current knowledge about the underlying environment is fully specified by the distribution *η*_*t*_. An unconstrained agent will attempt to use this knowledge and explore to further acquire information that helps identify an optimal action *A*^⋆^ ∈ $\underset{a\in 𝒜}{\arg\;\max}$ *Q*^⋆^(*H*_*t*_, *a*). By default, however, a capacity-limited agent may not be capable of obtaining all 𝕀_*t*_(𝓔; *A*^⋆^) bits of information from the world to learn such an optimal action *A*^⋆^. To remedy this, it behooves the agent to first determine an alternative learning target *χ* and then orient exploration to prioritize information gathering about this feasible surrogate. Naively discarding bits of information in each time period to obtain an easily learned target with small 𝕀_*t*_(𝓔; *χ*), however, may result in agent that is entirely unproductive with respect to the task at hand. Thus, while a good target *χ* does allow an agent to get away with exploring for less information, some bits have more utility to the task than others.

Rate-distortion theory (Berger, 1971; Shannon, 1959) is a branch of information theory (Cover & Thomas, 2012; Shannon, 1948) dedicated to the study of lossy compression problems which necessarily must optimize for a balance between the raw amount of information retained in the compression and the utility of those bits for some downstream task; a classic example of this from the information-theory literature is image compression down to a smaller resolution (fewer bits of information) without overly compromising the visual acuity of the content (bounded distortion). A capacity-limited agent will take its current knowledge *η*_*t*_ as the information source to be compressed in each time period *t* ∈ [*T*]. The learning target *χ*(𝓔) ∈ 𝒜 can be interpreted as the result of lossy compression, characterized by a channel or conditional probability distribution *p*(*χ* | 𝓔) that maps a potential realization of the unknown environment 𝓔 ∈ Θ to a corresponding distribution over actions. For a given realization of the environment *θ* ∈ Θ, one should interpret *p*(*χ* | 𝓔 = *θ*) as an agent’s belief about which actions are desirable taking 𝓔 = *θ* as reality. Naturally, the amount of information used contained in the environment about this action that is not accounted for by the agent’s interactions *H*_*t*_ thus far is precisely quantified by the mutual information between these two random variables, 𝕀_*t*_(𝓔; *χ*), where the *t* subscript captures the dependence of the agent’s beliefs *η*_*t*_ on the current random history *H*_*t*_.

Aside from identifying the data to be compressed, a lossy compression problem also requires the specification of a loss or distortion function *d* : 𝒜 × Θ → ℝ_≥0_ which helps to distinguish between target-relevant bits of information contained in the environment. Intuitively, without yet giving a precise mathematical definition of a distortion function, environment-target pairs yielding high distortion are commensurate with achieving high loss with respect to the task at hand. Thus, a good choice of learning target is one that can avoid large expected distortion, 𝔼_*t*_[*d*(*χ*, 𝓔)]. Putting these two pieces together, the fundamental limit of lossy compression is given by the rate-distortion function

$${𝓡}_{t}\left(D\right)=\underset{p\left(\chi |𝓔\right)}{\inf}{𝕀}_{t}\left(𝓔;\chi \right)\;\text{such}\;\text{that}\;{𝔼}_{t}\left[d\left(\chi ,𝓔\right)\right]\leq D,$$ (2)

which quantifies the absolute minimum amount of information needed from the environment to ensure expected distortion does not exceed a threshold *D* ∈ ℝ_≥0_. As an agent’s beliefs about the environment 𝓔 vary with time *η*_*t*_, it is natural for a capacity-limited agent to update its target over time as data accumulates. Accordingly, we denote the conditional distribution that achieves this infimum as *δ*_*t*_($\tilde{A}$_*t*_ | 𝓔) where $\tilde{A}$_*t*_ is the random variable representing the particular learning target or *target action* that achieves the rate-distortion limit in time period *t* (Equation 2). Some well-known, useful facts of the rate-distortion function are as follows:

**Fact 1** (Lemma 10.4.1 [Cover & Thomas, 2012]). *For all t* ∈ [*T*], *the rate-distortion function* 𝓡_*t*_(*D*) *is a non-negative, convex, and non-increasing function in D* ≥ 0.

A bounded decision maker with limited information processing can only hope to make near-optimal decisions. Thus, a natural way to define distortion is given by the expected performance shortfall between an optimal decision and the chosen one.

$$d\left(\tilde{a},\theta \right)={𝔼}_{t}\left[Q^{⋆}\left(H_{t},A^{⋆}\right)-Q^{⋆}(H_{t},\tilde{a})|𝓔=\theta \right].$$

The distortion threshold *D* ∈ ℝ_≥0_ input to the rate-distortion function is a free parameter specified by an agent designer that communicates a preferences for the minimization of rate versus the minimization of distortion; alternatively, one might hypothesize that this threshold is adapted within biological decision-making agents based on evolutionary pressures. In either case, this aligns with a perspective that a capacity-limited decision-making agent, while likely incapable of recovering optimal behavior, still aims to act productively with respect to the task at hand. If one is willing to tolerate significant errors and large amounts of regret, than decision-making should be far simpler in the sense that very few bits of information from the environment are needed to learn a suitable target action. Conversely, as prioritizing near-optimal behavior becomes more important, each decision requires greater cognitive effort as measure by the amount of information an agent must gather from the environment to learn $\tilde{A}$_*t*_. The power of rate-distortion theory, in part, lies in the ability to give precise mathematical form to this intuitive narrative, as demonstrated by an immediate consequence of Fact 1 for any *D* > 0,

$${𝕀}_{t}\left(𝓔;{\tilde{A}}_{t}\right)={𝓡}_{t}\left(D\right)\leq {𝓡}_{t}\left(0\right)\leq {𝕀}_{t}\left(𝓔;A^{⋆}\right)={ℍ}_{t}\left(A^{⋆}\right)-\underset{\geq 0}{\underbrace{{ℍ}_{t}\left(A^{⋆}|𝓔\right)}}\leq {ℍ}_{t}\left(A^{⋆}\right),$$

confirming that the amount of information needed to determine $\tilde{A}$_*t*_, in any time period, is less than what would be needed to identify an optimal action *A*^⋆^. Consequently, the exploration challenge faced by a capacity-limited decision-maker pursuing $\tilde{A}$_*t*_ in each time period is strictly easier than that of *A*^⋆^.

Alternatively, in lieu of presuming that an agent is cognizant of what constitutes a “good enough” solution, one may instead adopt the perspective that an agent is made aware of its own capacity limitations. In this context, agent capacity refers to a bound *R* ∈ ℝ_≥0_ on the number of bits an agent may hope to obtain from its interactions within the environment through exploration. While the rate-distortion function quantifies the minimum achievable rate subject to an expected distortion constraint, the distortion-rate function quantifies the minimum achievable expected distortion subject to a rate constraint:

$${𝒟}_{t}\left(R\right)=\underset{p\left(\chi |𝓔\right)}{\inf}{𝔼}_{t}\left[d\left(\chi ,𝓔\right)\right]\;\text{such}\;\text{that}\;{𝕀}_{t}\left(𝓔;\chi \right)\leq R.$$ (3)

Natural limitations on a decision-maker’s time or computational resources can be translated and expressed as limitations on the sheer amount of information *R* that can possibly be learned about a target action from interacting with the environment 𝓔. Moreover, the distortion-rate function 𝒟_*t*_(*R*) in any time period *t* obeys the identical properties of 𝓡_*t*_(*D*) outlined in Fact 1, such that agents with greater capacity are capable of achieving lower levels of expected distortion. It is oftentimes convenient that the rate-distortion function and distortion-rate function are inverses of one another such that 𝓡_*t*_(𝒟_*t*_(*R*)) = *R*.

In this section, we have provided a mathematical formulation for how a capacity-limited agent discerns *what to learn* in each time period so as to limit overall cognitive load in an information-theoretically optimal fashion while incurring bounded sub-optimality. Notably, we have yet to discuss how such an agent ultimately selects actions so as to facilitate efficient learning of the target action $\tilde{A}$_*t*_ computed via rate-distortion theory. To elucidate this, we dedicate the next section to the simple yet illustrative multi-armed bandit problem, which allows for theoretical and as well as empirical analysis.

### Multi-Armed Bandit

In this section, we begin with the formal specification of a multi-armed bandit problem (Bubeck & Cesa-Bianchi, 2012; Lai & Robbins, 1985; Lattimore & Szepesvári, 2020) before revisiting Thompson Sampling as a quintessential algorithm for identifying optimal actions. We then present a corresponding generalization of Thompson Sampling that takes an agent’s capacity limitations into account.

#### Problem Formulation.

We obtain a bandit environment as a special case of the problem formulation given in Continual Learning section by treating the initial observation as null *O*_0_ = ∅ while each subsequent observation denotes a reward signal *R*_*t*_ ∼ *ρ*(· | *A*_*t*_) drawn from an observation function *ρ* : 𝒜 → Δ(ℝ) that only depends on the most recent action selection *A*_*t*_ and not the current history *H*_*t*_ = (*A*_1_, *R*_1_, *A*_2_, *R*_2_, …, *A*_*t*−1_, *R*_*t*−1_). While the actions 𝒜 and total time periods *T* ∈ ℕ are known to the agent, the underlying reward function *ρ* is unknown and, consequently, the environment 𝓔 is itself a random variable such that *p*(*R*_*t*_ | 𝓔, *A*_*t*_) = *ρ*(*R*_*t*_ | *A*_*t*_). We let $\bar{\rho }$ : 𝒜 → [0, 1] denote the mean reward function $\bar{\rho }$(*a*) = 𝔼[*R*_*t*_ | *A*_*t*_ = *a*, 𝓔], ∀*a* ∈ 𝒜, and define an optimal action *A*^⋆^ ∈ $\underset{a\in 𝒜}{\arg\;\max}\bar{\rho }$ (*a*) as achieving the maximal mean reward denoted as *R*^⋆^ = $\bar{\rho }$ (*A*^⋆^), both of which are random variables due to their dependence on 𝓔.

Observe that, if the agent knew the underlying environment 𝓔 exactly, there would be no uncertainty in the optimal action *A*^⋆^; consequently, it is the agent’s epistemic uncertainty (Der Kiureghian & Ditlevsen, 2009) in 𝓔 that drives uncertainty in *A*^⋆^. Since learning is a process of acquiring information, an agent explores to learn about the environment and reduce this uncertainty. As there is only a null history at the start *H*_1_ = ∅, initial uncertainty in the environment 𝓔 ∈ Θ is given by the prior probabilities *η*_1_ ∈ Δ(Θ) while, as time unfolds, updated knowledge of the environment is reflected by posterior probabilities *η*_*t*_ ∈ Δ(Θ).

The customary goal within a multi-armed bandit problem is to identify an optimal action *A*^⋆^ and, in the next section, we review one such algorithm that is widely used in practice before motivating consideration of satisficing solutions for bandit problems.

#### Thompson Sampling & Satisficing.

As previously mentioned, standard choice of algorithm for identifying optimal actions in multi-armed bandit problems is Thompson Sampling (TS) (Russo et al., 2018; Thompson, 1933), which has been well-studied both theoretically (Agrawal & Goyal, 2012, 2013; Auer et al., 2002; Bubeck & Liu, 2013; Russo & Van Roy, 2016) and empirically (Chapelle & Li, 2011, Gopalan et al., 2014; Granmo, 2010; Scott, 2010). For convenience, we provide generic pseudocode for classic TS as Algorithm 3, whereas more granular classes of bandit problems (Bernoulli bandits or Gaussian bandits, for example) can often lead to more computationally explicit versions of TS that leverage special structure like conjugate priors (see (Russo et al., 2018) for more detailed implementations). In each time period *t* ∈ [*T*], a TS agent proceeds by drawing one sample *θ*_*t*_ ∼ *η*_*t*_(𝓔), representing a statistically-plausible hypothesis about the underlying environment based on the agent’s current posterior beliefs from observing the history *H*_*t*_; the agent then proceeds as if this sample dictates reality and acts optimally with respect to it, drawing an action to execute this time period *A*_*t*_ uniformly at random among the optimal actions for this realization of 𝓔 = *θ*_*t*_ of the environment. Executing actions in this manner recovers the hallmark probability-matching principle (Russo & Van Roy, 2016; Scott, 2010) of classic TS whereby, in each time period *t* ∈ [*T*], the agent selects actions according to their (posterior) probability of being optimal given everything observed up to this point in *H*_*t*_ or, more formally, *π*_*t*_(*a*) = *p*_*t*_(*A*^⋆^ = *a*), ∀*a* ∈ 𝒜.


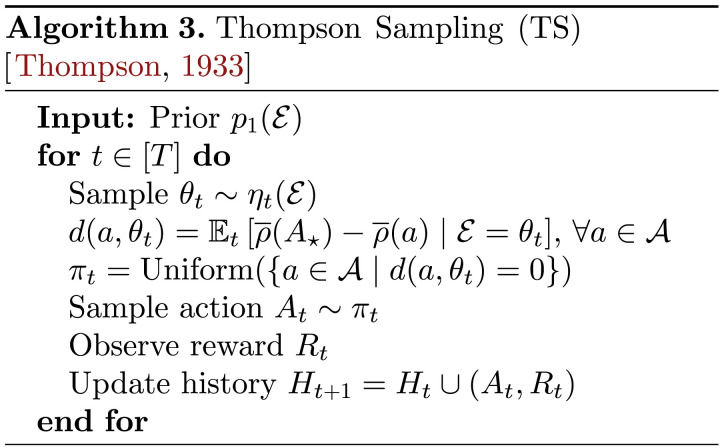


Naturally, a core premise of this work is to consider decision-making problems where an agent’s inherent and unavoidable capacity limitations drastically impact the tractability of learning optimal actions. While there are other classes of algorithms for handling multi-armed bandit problems (Auer et al., 2002; Powell & Ryzhov, 2012; Russo & Van Roy, 2014, 2018a; Ryzhov et al., 2012), TS serves an exemplary representative among them that relentlessly pursues the optimal action *A*^⋆^, by design. Consider a human decision maker faced with a bandit problem containing 1,000,000,000 (one trillion) arms—does one genuinely expect any individual to successfully identify *A*^⋆^ within a reasonable amount of time? Similarly, the Bayesian regret bound for TS scales with the agent’s prior entropy in *A*^⋆^ (Russo & Van Roy, 2016), informing us that the performance shortfall of TS will increase as the number of actions tends to ∞.

Satisficing is a longstanding, well-studied idea about how to understand resource-limited cognition (Newell et al., 1958; Newell & Simon, 1972; Simon, 1955, 1956, 1982) in which an agent settles for the first recovered solution that is deemed to be “good enough,” for some suitable notion of goodness. Inspired by this idea, Russo and Van Roy (2018b, 2022) present the Satisficing Thompson Sampling (STS) algorithm, which we present as Algorithm 4, to address the shortcomings of algorithms like TS that relentlessly pursue *A*^⋆^. STS employs a minimal adjustment to the original TS algorithm through a threshold parameter *ε* ≥ 0, which an agent designer may use to communicate that identifying a *ε*-optimal action would be sufficient for their needs. The use of a minimum over all such *ε*-optimal actions instead of a uniform distribution reflects the idea of settling for the first solution deemed to be “good enough” according to *ε*. Naturally, the intuition follows that as *ε* increases and the STS agent becomes more permissive, such *ε*-optimal actions can be found in potentially far fewer time periods than what is needed to obtain *A*^⋆^ through TS. If we define an analogous random variable to *A*^⋆^ as *A*_*ε*_ ∼ min({*a* ∈ 𝒜 | 𝔼_*t*_[$\bar{\rho }$(*A*^⋆^) − $\bar{\rho }$(*a*) | 𝓔 = *θ*_*t*_] ≤ *ε*}) then STS simply employs probability matching with respect to this alternative target as *π*_*t*_(*a*) = *p*_*t*_(*A*_*ε*_ = *a*), ∀*a* ∈ 𝒜 and, as *ε* ↓ 0, recovers TS as a special case. Russo and Van Roy (2022) go on to prove a complementary information-theoretic regret bound for STS, which depends on the mutual information between the environment and *A*_*ε*_, 𝕀_1_(𝓔; *A*_*ε*_), rather than the prior entropy in the optimal action *A*^⋆^, ℍ_1_(*A*^⋆^).


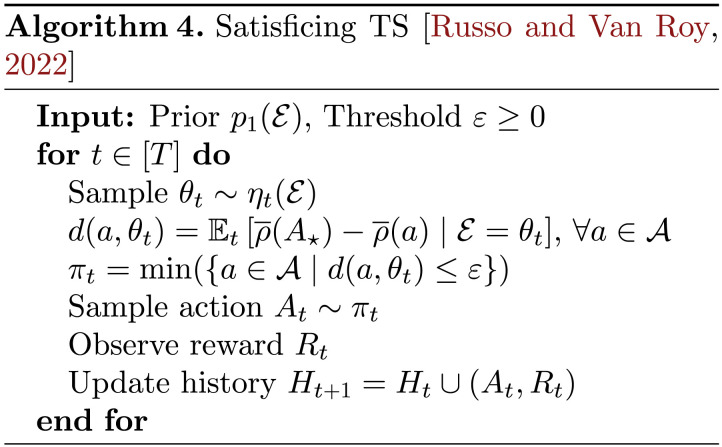


While it is clear that STS does embody the principle of satisficing for a capacity-limited decision maker, the *A*_*ε*_ action targeted by a STS agent instead of *A*^⋆^ only achieves some arbitrary and unspecified trade-off between the simplicity of what the agent set out to learn and the utility of the resulting solution, as *ε* varies. Rather than setting for an arbitrary balance between these competing concerns, the next section examines how rate-distortion theory yields a target action that strikes the best trade-off.

#### Rate-Distortion Theory for Target Actions.

The notion of a target action is based on the observation that *A*^⋆^ = *f*(𝓔) is merely a statistic of the environment whose computation is determined by some function *f*. It follows that a surrogate action an agent may alternatively prioritize during learning will be some other computable statistic of the environment that embodies a kind of trade-off between two key properties: (1) ease of learnability and (2) bounded sub-optimality or performance shortfall relative to *A*^⋆^.

The previous section already gives two concrete examples of potential target actions, *A*^⋆^ and *A*_*ε*_, where the former represents an extreme point on the spectrum of potential learning targets as one that demands a potentially intractable amount of information to identify but comes with no sub-optimality. At the other end of the spectrum, there is simply the uniform random action $\bar{A}$ ∼ Uniform(𝒜) which requires no learning or sampling on the part of the agent to learn it but, in general, will likely lead to considerably large performance shortfall relative to an optimal solution. While, for any fixed *ε* > 0, *A*_*ε*_ lives in between these extremes, it also suffers from two shortcomings of its own. Firstly, by virtue of satisficing and a willingness to settle for anything that is “good enough,” it is unclear how well *A*_*ε*_ balances between the two aforementioned desiderata. In particular, the parameterization of *A*_*ε*_ around *ε* as an upper bound to the expected regret suggests that there could exist an even simpler target action which is also *ε*-optimal but easier to learn insofar as it requires the agent obtain fewer bits of information from the environment. Secondly, from a computational perspective, a STS agent striving to learn *A*_*ε*_ (just as a TS agent does for learning *A*^⋆^) computes the same statistic repeatedly across all *T* time periods. Meanwhile, with every step of interaction, the agent’s knowledge of the environment 𝓔 is further refined, potentially changing the outlook on what can be tractably learned in subsequent time periods. This would suggest that one may stand to have considerable performance gains by designing agents that adapt their learning target as knowledge of the environment accumulates, rather than iterating on the same static computation. From a biological view, this encapsulates a perspective that an organism’s outlook on learning goals adapts with its knowledge of the world.

Arumugam and Van Roy (2021a) leverage the following rate-distortion function and use the resulting learning target $\tilde{A}$_*t*_ ∼ *δ*_*t*_(· | 𝓔) in each time period as a dynamic replacement of the static *A*^⋆^ or *A*_*ε*_ in TS and STS, respectively.

$${𝓡}_{t}\left(D\right)=\underset{p\left(\tilde{A}|𝓔\right)}{\inf}{𝕀}_{t}\left(𝓔;\tilde{A}\right)\;\text{such}\;\text{that}\;{𝔼}_{t}\left[d\left(\tilde{A},𝓔\right)\right]\leq D.$$ (4)

In order to satisfy the second desideratum of bounded performance shortfall for learning targets and to facilitate a regret analysis, Arumugam and Van Roy (2021a) define the distortion function as the expected squared regret of the given action for the given realization of the environment:

$$d\left(\tilde{a},\theta \right)={𝔼}_{t}\left[{\left(\bar{\rho }\left(A^{⋆}\right)-\bar{\rho }\left(\tilde{a}\right)\right)}^{2}|𝓔=\theta \right].$$

While having bounded expected distortion satisfies our second criterion for a learning target, the fact that $\tilde{A}$_*t*_ requires fewer bits of information to learn is immediately given by properties of the rate-distortion function 𝓡_*t*_(*D*) itself, through Fact 1. We present Rate-Distortion Thompson Sampling (RDTS) as Algorithm 5, representing an agent that performs probability matching with respect to $\tilde{A}$_*t*_ in each time period, given an input distortion threshold *D* ∈ ℝ_≥0_. In Appendix C, we offer a theoretical analysis of RDTS via an upper bound on Bayesian regret expressed as a sum of two terms: one term depending on 𝓡_1_(*D*) to characterize the regret incurred learning $\tilde{A}$_*t*_ and another term dependent on *D* that expresses the sub-optimality of pursuing $\tilde{A}$_*t*_ instead of *A*^⋆^. Using the fact that the rate-distortion function 𝓡_*t*_(*D*) and distortion-rate function 𝒟_*t*_(*R*) have an inverse relationship, a corollary of this result yields a capacity-sensitive performance guarantee that depends on an agent’s capacity limit *R* ∈ ℝ_≥0_ and the distortion-rate function 𝒟_1_(*R*).


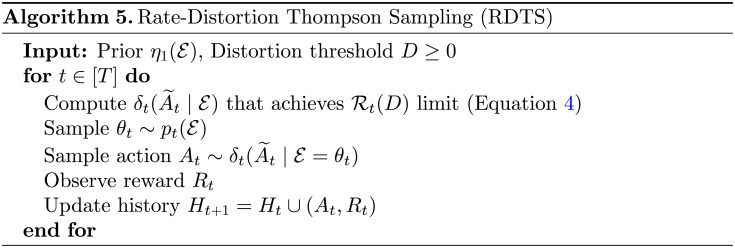


#### Experiments.

In order to make the algorithm of the previous section (Algorithm 5) amenable to practical implementation, Arumugam and Van Roy (2021a) look to the classic Blahut-Arimoto algorithm (Arimoto, 1972; Blahut, 1972). Just as TS and STS perform probability matching with respect to *A*^⋆^ and *A*_*ε*_ in each time period, respectively, the Blahut-Arimoto STS (BLASTS) algorithm (presented as Algorithm 2 where one should recall that reward maximization and regret minimization are equivalent) conducts probability matching with respect to $\tilde{A}$_*t*_ in each time period to determine the policy: *π*_*t*_(*a*) = *p*_*t*_($\tilde{A}$_*t*_ = *a*), ∀*a* ∈ 𝒜. For two discrete random variables representing an uncompressed information source and the resulting lossy compression, the Blahut-Arimoto algorithm computes the channel that achieves the rate-distortion limit (that is, achieve the infimum in Equation 4) by iterating alternating update equations until convergence. More concretely, the algorithm is derived by optimizing the Lagrangian of the constrained optimization (Boyd & Vandenberghe, 2004) that is the rate-distortion function, which is itself known to be a convex optimization problem (Chiang & Boyd, 2004). We refer readers to Arumugam and Van Roy (2021a) for precise computational details of the Blahut-Arimoto algorithm for solving the rate-distortion function 𝓡_*t*_(*D*) that yields $\tilde{A}$_*t*_ as well as Arumugam and Van Roy (2021b) for details on the exact theoretical derivation.

One salient detail that emerges from using the Blahut-Arimoto algorithm in this manner is that it no longer depends on a distortion threshold *D* ∈ ℝ_≥0_ as input but, instead, provides a value of the Lagrange multiplier *β* ∈ ℝ_≥0_; lower values of *β* communicate a preferences for rate minimization whereas larger values of *β* prioritize distortion minimization. To each value of *β*, there is an associate distortion threshold *D* as *β* represents the desired slope achieved along the corresponding rate-distortion curve (Blahut, 1972; Csiszár, 1974a, 1974b). As, in practice, *η*_*t*_(𝓔) tends to be a continuous distribution, Arumugam and Van Roy (2021a) induce a discrete information source by drawing a sufficiently large number of Monte-Carlo samples and leveraging the resulting empirical distribution, which is a theoretically-sound estimator of the true rate-distortion function (Harrison & Kontoyiannis, 2008; Palaiyanur & Sahai, 2008).

**Figure 4.**
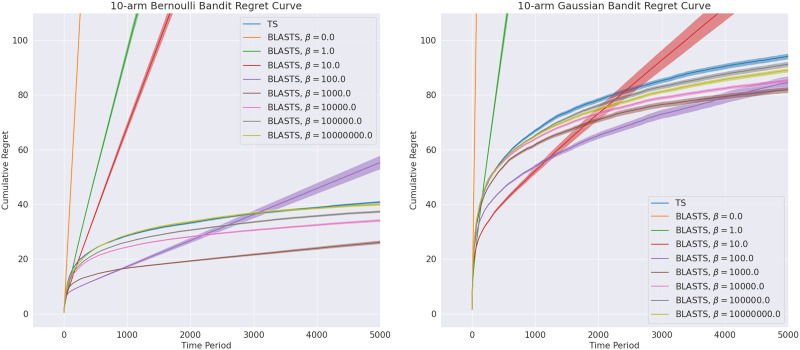
Cumulative regret curves for Bernoulli and Gaussian bandits with 10 independent arms comparing traditional Thompson Sampling (TS) against Blahut-Arimoto STS (BLASTS), sweeping over the *β* hyperparameter of the latter.

As these target actions {$\tilde{A}$_*t*_}_*t*∈[*T*]_ are born out of a need to balance the simplicity and utility of what an agent aims to learn from its interactions within the environment, we can decompose empirical results into those that affirm these two criteria are satisfied in isolation. Since assessing utility or, equivalently, performance shortfall is a standard evaluation metric used throughout the literature, we begin there and offer regret curves in Figure 4 for Bernoulli and Gaussian bandits with 10 independent arms (matching, for example, the empirical evaluation of Russo and Van Roy [2018a]); recall that the former implies Bernoulli rewards *R*_*t*_ ∼ Bernoulli($\bar{\rho }$(*A*_*t*_)) while the latter yields Gaussian rewards with unit variance *R*_*t*_ ∼ 𝒩($\bar{\rho }$(*A*_*t*_), 1). For readers unfamiliar with such plots, recall that the regret in a given time period reflects the performance shortfall between an agent’s chosen action and the optimal action. Cumulative regret curves as shown in Figure 4 show the sum of all per-period regret up to and including the current time period. A sub-optimal agent will yield linear regret where the slope conveys the degree of the sub-optimality. Meanwhile, optimal agents will eventually incur per-period regret of zero and so will have cumulative regret that eventually converges to a fixed value. We evaluate TS and BLASTS agents where, for the latter, the Lagrange multiplier hyperparameter *β* ∈ ℝ_≥0_ is fixed and tested over a broad range of values. All agents begin with a Beta(1, 1) prior for each action of the Bernoulli bandit and a 𝒩(0, 1) prior for the Gaussian bandit. For each individual agent, the cumulative regret incurred by the agent is plotted over each time period *t* ∈ [*T*].

Recalling that our distortion function is directly connected to the expected regret of the BLASTS agent, we observe that smaller values of *β* so aggressively prioritize rate minimization that the resulting agents incur linear regret; in both bandit environments, this trend persists for all values *β* ≤ 100. Notably, as *β* ↑ ∞, we observe the resulting agents yield performance more similar to regular TS. This observation aligns with expectations since, for a sufficiently large value of *β*, the Blahut-Arimoto algorithm will proceed to return a channel that only places probability mass on the distortion-minimizing actions, which are indeed, the optimal actions *A*^⋆^ for each realization of the environment. A notable auxiliary finding in these results, also seen in the original experiments of Arumugam and Van Roy (2021a), is that intermediate values of *β* manage to yield regret curves converging towards the optimal policy more efficiently that TS; this is, of course, only possible when the distortion threshold *D* implied by a particular setting of *β* falls below the smallest action gap of the bandit problem.

**Figure 5.**
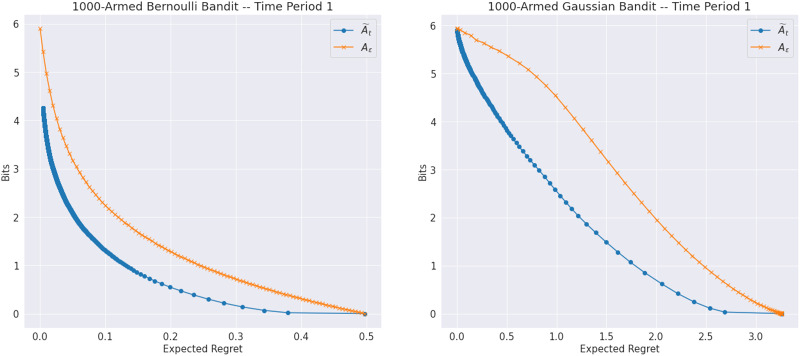
Rate-distortion curves for target actions computed via BLASTS ($\tilde{A}$_*t*_) and STS (*A*_*ε*_) in the first time periods of Bernoulli and Gaussian bandits with 1000 independent arms.

**Figure 6.**
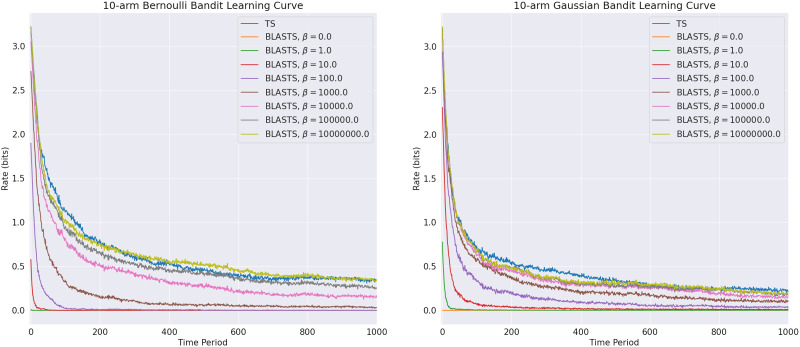
Rate curves for Bernoulli and Gaussian bandits with 10 independent arms comparing traditional Thompson Sampling (TS) against Blahut-Arimoto STS (BLASTS), sweeping over the *β* hyperparameter of the latter.

While the previous experiments confirm that BLASTS can be used to instantiate a broad spectrum of agents that target actions of varying utilities, it is difficult to assess the simplicity of these targets and discern whether or not less-performant target actions can in fact be identified more quickly than near-optimal ones. As a starting point, one might begin with the agent’s prior over the environment and compute 𝕀_1_(𝓔; $\tilde{A}$_*t*_) to quantify how much information each agent’s initial learning target requires from the environment *a priori*. In Figure 5, we compare this to 𝕀_1_(𝓔; *A*_*ε*_) and sweep over the respective *β* and *ε* values to generate the result rate-distortion curves for Bernoulli and Gaussian bandits with 1000 independent arms. The results corroborate earlier discussion of how a STS agent engages with a learning target *A*_*ε*_ that yields *some* trade-off between ease of learnability and performance, but not necessarily the best trade-off. In contrast, since 𝓡_1_(*D*) ≈ 𝕀_1_(𝓔; $\tilde{A}$_*t*_) (where the approximation is due to sampling), we expect and do indeed recover a better trade-off between rate and performance using the Blahut-Arimoto algorithm. To verify that target actions at the lower end of the spectrum (lower rate and higher distortion) can indeed by learned more quickly, we can plot the rate of the channel *δ*_*t*_($\tilde{A}$_*t*_ | 𝓔) computed by BLASTS across time periods, as shown in Figure 6; for TS, we additionally plot the entropy over the optimal action ℍ_*t*_(*A*^⋆^) as time passes and observe that smaller values of *β* lead to learning targets with smaller initial rates that decay much more quickly than their counterparts at larger values of *β*. Again, as *β* ↑ ∞, these rate curves concentrate around that of regular TS.

Overall, this section has provided an overview of prior work that moves past the standard goal of finding optimal actions *A*^⋆^ in multi-armed bandit problems and towards capacity-limited decision-making agents. Extending beyond the empirical findings observed in these prior works, we provide additional experiments (see Figure 6) that show how the minimization of rate leads to target actions that are simpler to learn, allowing for an agent to curtail its interactions with the environment in fewer time periods and respect limitations on time and computational resources. Crucially, rate-distortion theory emerges as a natural conduit for identifying target actions that balance between respecting an agent’s limitations while still being sufficiently useful for the task at hand.

## DISCUSSION

In this paper, we have introduced capacity-limited Bayesian reinforcement learning, capturing a novel perspective on lifelong learning under a limited cognitive load while also surveying existing theoretical and algorithmic advances specific to multi-armed bandits (Arumugam & Van Roy, 2021a) and reinforcement learning (Arumugam & Van Roy, 2022). Taking a step back, we now situate our contributions in a broader context by reviewing related work on capacity-limited cognition as well as information-theoretic reinforcement learning. As our framework sits at the intersection of Bayesian inference, reinforcement learning, and rate-distortion theory, we use this opportunity to highlight particularly salient pieces of prior work that sit at the intersection Bayesian inference and rate-distortion theory as well as the intersection of reinforcement learning and rate-distortion theory, respectively. Furthermore, while the algorithms discussed in this work all operationalize the Blahut-Arimoto algorithm and Thompson Sampling as the primary mechanisms for handling rate-distortion optimization and exploration respectively, we also discuss opportunities to expand to more sophisticated strategies for computing a target action and exploring once it has been determined. Lastly, we conclude our discussion by returning to a key assumption used throughout this work that an agent consistently maintains idealized beliefs about the environment 𝓔 through perfect Bayesian inference.

### Related Work on Learning, Decision-Making, and Rate-Distortion Theory

There is a long, rich literature exploring the natural limitations on time, knowledge, and cognitive capacity faced by human (and animal) decision makers (Amir et al., 2020; Bhui et al., 2021; Binz & Schulz, 2022; Brown et al., 2022; Gershman et al., 2015; Gigerenzer & Goldstein, 1996; Griffiths et al., 2015; Ho et al., 2022; Icard & Goodman, 2015; Lieder & Griffiths, 2020; Newell & Simon, 1972; Newell et al., 1958; Prystawski et al., 2022; Simon, 1956, 1982; Shugan, 1980; Vul et al., 2014). Crucially, our focus is on a recurring theme throughout this literature of modeling these limitations on cognitive capabilities as being information-theoretic in nature (Bari & Gershman, 2022; Botvinick et al., 2015; Gershman, 2020, 2023; Gershman & Lai, 2020; Ho et al., 2020; Jakob & Gershman, 2022; Lai & Gershman, 2021; Mikhael et al., 2021; Parush et al., 2011; Peng, 2005; Sims, 2003, 2016, 2018; Zénon et al., 2019).

Broadly speaking and under the episodic reinforcement learning formulation of Appendix B, these approaches all center around the perspective that a policy *π*_*h*_ : 𝒮 → Δ(𝒜) mapping states to distributions over actions should be modeled as a communication channel that, like a human decision-maker with limited information processing capability, is subject to a constraint on the maximal number of bits that may be transmitted across it. Consequently, an agent aspiring to maximize returns must do so subject to this constraint on policy complexity; conversely, an agent ought to transmit the minimum amount of information possible while it endeavors to reach a desired level of performance (Polani, 2009, 2011; Rubin et al., 2012; Tishby & Polani, 2011). Paralleling the distortion-rate function 𝒟(*R*), the resulting policy-optimization objective follows as $\underset{\pi \in {\left\{𝒮\to \Delta \left(𝒜\right)\right\}}^{H}}{\sup}$ 𝔼[*Q*^*π*^(*S*, *A*)] such that 𝕀(*S*; *A*) ≤ *R*. It is important to acknowledge that such a formulation sits directly at the intersection of reinforcement learning and rate-distortion theory without invoking any principles of Bayesian inference. Depending on the precise work, subtle variations on this optimization problem exist from choosing a fixed state distribution for the random variable *S* (Polani, 2009, 2011), incorporating the state visitation distribution of the policy being optimized (Gershman, 2020; Lai & Gershman, 2021; Still & Precup, 2012), or assuming access to the generative model of the MDP and decomposing the objective across a finite state space (Rubin et al., 2012; Tishby & Polani, 2011). In all of these cases, the end empirical result tends to converge by also making use of variations on the classic Blahut-Arimoto algorithm to solve the Lagrangian associated with the constrained optimization (Boyd & Vandenberghe, 2004) and produce policies that exhibit higher entropy across states under an excessively limited rate *R*, with a gradual convergence towards the greedy optimal policy as *R* increases.

The alignment between this optimization problem and that of the distortion-rate function is slightly wrinkled by the non-stationarity of the distortion function (here, *Q*^*π*^ is used as an analogue to distortion which changes as the policy or channel does) and, when using the policy visitation distribution for *S*, the non-stationarity of the information source. Despite these slight, subtle mismatches with the core rate-distortion problem, the natural synergy between cognitive and computational decision making (Lake et al., 2017; Tenenbaum et al., 2011) has led to various reinforcement-learning approaches that draw direct inspiration from this line of thinking (Abel et al., 2019; Goyal, Bengio, et al., 2020; Goyal, Sodhani, et al., 2020; Goyal et al., 2019; Klyubin et al., 2005; Lerch & Sims, 2018, 2019; Ortega & Braun, 2011, 2013; Shafieepoorfard et al., 2016; Still & Precup, 2012; Tiomkin & Tishby, 2017), most notably including parallel connections to work on “control as inference” or KL-regularized reinforcement learning (Fox et al., 2016; Galashov et al., 2019; Haarnoja et al., 2017, 2018; Kappen et al., 2012; Levine, 2018; Tirumala et al., 2019; Todorov, 2007; Toussaint, 2009; Ziebart, 2010). Nevertheless, despite their empirical successes, such approaches lack principled mechanisms for addressing the exploration challenge (O’Donoghue et al., 2020). In short, the key reason behind this is that the incorporation of Bayesian inference allows for a separation of reducible or epistemic uncertainty that exists due to an agent’s lack of knowledge versus irreducible or aleatoric uncertainty that exists due to the natural stochasticity that may exist within a random outcome (Der Kiureghian & Ditlevsen, 2009). Without leveraging a Bayesian setting, a random variable denoting an agent’s belief about the environment 𝓔 or underlying MDP 𝓜^⋆^ no longer exists and a channel like the ones explored throughout this work from beliefs to action cease to exist. That said, the notion of rate preserved by these methods has been shown to constitute a reasonable notion of policy complexity (Lai & Gershman, 2021) and future work may benefit from combining the two approaches.

Similar to human decision making (Gershman, 2018, 2019; Schulz & Gershman, 2019), provably-efficient reinforcement-learning algorithms have historically relied upon one of two possible exploration strategies: optimism in the face of uncertainty (Auer et al., 2009; Azar et al., 2017; Bartlett & Tewari, 2009; Brafman & Tennenholtz, 2002; Dann & Brunskill, 2015; Dann et al., 2017; Dong et al., 2022; Jaksch et al., 2010; Jin et al., 2018; Kakade, 2003; Kearns & Singh, 2002; Strehl et al., 2009; Zanette & Brunskill, 2019) or posterior sampling (Agrawal & Jia, 2017; Lu & Van Roy, 2019; Lu et al., 2023; Osband et al., 2013; Osband & Van Roy, 2017). While both paradigms have laid down solid theoretical foundations, a line of work has demonstrated how posterior-sampling methods can be more favorable both in theory and in practice (Dwaracherla et al., 2020; Osband, Blundell, et al., 2016; Osband, Van Roy, et al., 2016; Osband et al., 2013, 2019; Osband & Van Roy, 2017). The theoretical results discussed in this work advance and further generalize this line of thinking through the concept of learning targets, introduced by Lu et al. (2023), which open up new avenues for entertaining solutions beyond optimal policies and conditioning an agent’s exploration based on what it endeavors to learn from its environment; future work may be able to draw a tangential but interesting parallel between such exploratory strategies and, for example, those empirically observed in preschool children (Cook et al., 2011) who are demonstrably capable of designing interventions targeted towards maximizing information gain about particular facets of the environment. While this literature traditionally centers on consideration of a single agent interacting within its environment, generalizations to multiple agents acting concurrently while coupled through shared beliefs have been formalized and examined in theory as well as in practice (Chen et al., 2022; Dimakopoulou & Van Roy, 2018; Dimakopoulou et al., 2018); translating the ideas discussed here to further account for capacity limitations in that setting constitutes a promising direction for future work.

Finally, we note while the work cited thus far was developed in the reinforcement learning community, the coupling of rate-distortion theory and Bayesian inference to strike a balance between the simplicity and utility of what an agent learns has been studied extensively by Gottwald and Braun (2019), who come from an information-theoretic background studying bounded rationality (Ortega & Braun, 2011, 2013). Perhaps the key distinction between the work surveyed here and theirs is the further incorporation of reinforcement learning, which then provides a slightly more precise foundation upon which existing machinery can be repurposed to derive theoretical results like regret bounds. In contrast, the formulation of Gottwald and Braun (2019) follows more abstract utility-theoretic decision making while also leveraging ideas from microeconomics and generalizing beyond from standard Shannon information-theoretic quantities; we refer readers to their excellent, rigorous treatment of this topic.

### Generalizations to Other Families of Decision Rules

The previous sections demonstrated several concrete implementations of capacity-limited Bayesian decision-making. We focused on BLASTS, an algorithm that generalizes Thompson Sampling, which itself is already a quintessential algorithm for navigating the explore-exploit tradeoff in a principled manner in multi-armed bandit and sequential decision-making problems. That said, however, we emphasize that BLASTS is only one particular instantiation of the framework espoused by the rate-distortion function of Equation 2. Here, we briefly sketch other directions in which the framework has been or could be applied.

First, the general framework of capacity-limited Bayesian decision-making can, in principle, be applied to any algorithm that, when supplied with beliefs about the environment and a particular target for learning, induces a policy to execute in the environment. For example, in *information-directed sampling*, choices are made not only based on current beliefs about immediate rewards but also based on how actions produce informative consequences that can guide future behavior (Hao & Lattimore, 2022; Hao et al., 2022; Lu et al., 2023; Russo & Van Roy, 2014, 2018a). This strategy motivates a decision-maker to engage in *direct exploration* as opposed to *random exploration* (Thompson Sampling being one example) (Wilson et al., 2014) and better resolve the explore-exploit dilemma. Work by Arumugam and Van Roy (2021b) has extended the BLASTS algorithm to develop variants of information-directed sampling that similarly minimize the rate between environment estimates and actions. Future work could explore even richer families of decision-rules such as those based on Bayes-optimal solutions over longer time horizons (Duff, 2002) and even ones that look past the KL-divergence as the core quantifier of information (Lattimore & Gyorgy, 2021; Lattimore & Szepesvári, 2019; Zimmert & Lattimore, 2019).

Additionally, BLASTS itself uses a seminal algorithm from the information-theory literature to ultimately address the rate-distortion optimization problem and find the decision-rule that optimally trades off reward and information—namely, the Blahut-Arimoto algorithm (Arimoto, 1972; Blahut, 1972). However, this standard algorithm, while mathematically sound for random variables taking values on abstract spaces (Csiszár, 1974b), can only be made computationally tractable in the face of discrete random variables. Extending to general *input* distributions (*e.g.*, distributions with continuous or countable support) occurs through the use of an estimator with elegant theoretical properties such as asymptotic consistency (Harrison & Kontoyiannis, 2008; Palaiyanur & Sahai, 2008). Despite this, it is still limited to *output* distributions that have finite support. This limits its applicability to problems where the action space is finite and relatively small (even if the environment space is complex). Thus, an important direction for future research will be to develop algorithms for finding capacity-limited decision-rules based on versions of Blahut-Arimoto designed for general output distributions (*e.g.*, particle filter-based algorithms [Dauwels, 2005]).

### Capacity-Limited Estimation and Alternative Information Bottlenecks

Throughout this paper, we have assumed that environment estimation is not directly subject to capacity-limitations and that decision-makers perform perfect Bayesian inference. Naturally, however, this idealized scenario isn’t guaranteed to hold for biological or artificial decision making agents. One high-level perspective on the core problem addressed in this work is that decision-making agents cannot acquire unbounded quantities of information from the environment—this reality motivates the need to prioritize information and rate-distortion theory emerges as a natural tool for facilitating such a prioritization scheme.

By the same token, capacity-limited decision-making agents should also seldom find themselves capable of *retaining* all bits of information uncovered about the underlying environment 𝓔. If this were possible, then maintaining perfect belief estimates about the environment via *η*_*t*_ would be a reasonable supposition. In reality, however, an agent must also be judicious in what pieces of environment information are actually retained. Lu et al. (2023) introduce terminology for discussing this limited corpus of world knowledge as an *environment proxy*, $\tilde{𝓔}$. The lack of fidelity between this surrogate and true environment 𝓔 translates to the approximate nature of an agent’s Bayesian inference when maintaining beliefs about $\tilde{𝓔}$ in lieu of 𝓔. For biological decision-making agents, the concept of a proxy seems intuitive, as noted by Herbert Simon (Simon, 1956) many decades ago: “we are not interested in describing some physically objective world in its totality, but only those aspects of the totality that have relevance as the ‘life space’ of the organism considered. Hence, what we call the ‘environment’ will depend upon the ‘needs,’ ‘drives,’ or ‘goals’ of the organism.”

Curiously, the relationship between the original environment 𝓔 and this proxy $\tilde{𝓔}$ can also be seen as a lossy compression problem where only a salient subset of the cumulative environment information need by retained by the agent for competent decision-making. Consequently, the associated rate-distortion function and the question of what suitable candidate notions of distortion apply may likely be an interesting object of study for future work. Practical optimization of such a rate-distortion function would likely benefit from recent statistical advances in empirical distribution compression (Dwivedi & Mackey, 2021) to permit representing the information source via a limited number of Monte-Carlo samples.

Finally, although an in-depth analysis of capacity-limits on inference is beyond the scope of the current paper, it is worth noting that recent findings in neuroscience support the possibility of a bottleneck on choice processes even if the bottleneck on inference is minimal. For example, when trained on stimuli presented at different angles, mice have been shown to discriminate orientations as low as 20°–30° based on *behavioral* measures (Abdolrahmani et al., 2019). However, direct *neural* measurements from visual processing regions reveal sensitivity to orientations as low as 0.37° (Stringer et al., 2021). The higher precision (nearly 100× higher) of sensory versus behavioral discrimination is consistent with a greater information bandwidth on inference compared to choice, as assumed in the current version of the model.^2^ Similarly, work tracking the development of decision-making strategies in children provides evidence of capacity limits on choice processes even in the absence of limits on inference. For example, Decker et al. (2016) report that on a task designed to dissociate model-free versus model-based learning mechanisms, 8–12 year olds show signs of encoding changes in transition structure (longer reaction times) but do not appear to use this information to make better decisions, unlike 13–17 year olds and adults.^3^ This result is consistent with a distinct bottleneck between inference and action that has a developmental trajectory. In short, the analyses developed in this paper provide a starting point for understanding the computational principles that underlie cases in which decision-makers display approximately optimal inference but systematically suboptimal choice.

### Conclusion

Our goal in this paper has been to review key insights from work on capacity-limited Bayesian decision-making by Arumugam and Van Roy (2021a, 2022) and situate it within existing work on capacity-limited cognition and decision-making. This discussion naturally leads to a number of questions, in particular, how the general framework presented can be applied to a wider range of algorithms, how other kinds of information bottlenecks could affect learning, and whether humans and other animals are capacity-limited Bayesian decision-makers. We hope that by formally outlining the different components of capacity-limited inference and choice, the current work can facilitate future cross-disciplinary investigations to address such topics.

## ACKNOWLEDGMENTS

We thank the action editor and reviewers for their helpful comments and feedback on the article.

## AUTHOR CONTRIBUTIONS

D.A.: Conceptualization; Formal analysis; Methodology; Writing – review & editing; M.K.H.: Conceptualization; Formal analysis; Methodology; Writing – review & editing; N.D.G: Conceptualization; Supervision; Writing – review & editing; B.V.R.: Conceptualization; Supervision; Writing – review & editing.

## FUNDING INFORMATION

Financial support from Army Research Office (ARO) grant W911NF2010055 (to BVR) is gratefully acknowledged.

## Notes

^1^ At the very first timestep, the initial history only consists of an initial observation *H*_0_ = *O*_0_ ∈ 𝒪.^2^ Special thanks to Harrison Ritz and Jonathan Cohen for pointing out the connection to these findings.^3^ Special thanks to Catherine Hartley for pointing out the connection to these findings.

## APPENDIX A: PRELIMINARIES

In this section, we provide details on our notation and information-theoretic quantities used throughout the paper. We encourage readers to consult (Cover & Thomas, 2012; Duchi, 2021; Gray, 2011; Polyanskiy & Wu, 2024) for more background on information theory. We define all random variables with respect to a probability space (Ω, 𝓕, ℙ). For any two random variables *X* and *Y*, we use the shorthand notation *p*(*X*) ≜ ℙ(*X* ∈ ·) to denote the law or distribution of the random variable *X* and, analogously, *p*(*X* | *Y*) ≜ ℙ(*X* ∈ · | *Y*) as well as *p*(*X* | *Y* = *y*) ≜ ℙ(*X* ∈ · | *Y* = *y*) for the associated conditional distributions given *Y* and a realization of *Y* = *y*, respectively. For the ease of exposition, **we will assume throughout this work that all random variables are discrete**; aside from there being essentially no loss of generality by assuming this (see Equation 2.2.1 of Duchi [2021] or Theorem 4.5 of Polyanskiy and Wu [2024] for the Gelfand-Yaglom-Perez definition of divergence [Gelfand & Yaglom, 1959; Perez, 1959]), extensions to arbitrary random variables taking values on abstract spaces are straightforward and any theoretical results presented follow through naturally to these settings. In the case of any mentioned real-valued or vector-valued random variables, one should think of these as discrete with support obtained from some suitably fine quantization such that the resulting discretization error is negligible. For any natural number *N* ∈ ℕ, we denote the index set as [*N*] ≜ {1, 2, …, *N*}. For any arbitrary set 𝒳, Δ(𝒳) denotes the set of all probability distributions with support on 𝒳. For any two arbitrary sets 𝒳 and 𝒴, we denote the class of all functions mapping from 𝒳 to 𝒴 as {𝒳 → 𝒴} ≜ {*f* | *f* : 𝒳 → 𝒴}.

We define the mutual information between any two random variables *X*, *Y* through the Kullback-Leibler (KL) divergence

$$𝕀\left(X;Y\right)=D_{\text{KL}}\left(p\left(X,Y\right)\|p\left(X\right)p\left(Y\right)\right),\;D_{\text{KL}}\left(q_{1}\|q_{2}\right)=\sum_{x\in 𝒳}q_{1}\left(x\right)\log\left(\frac{q_{1}\left(x\right)}{q_{2}\left(x\right)}\right),$$

where *q*_1_, *q*_2_ ∈ Δ(𝒳) are both probability distributions. An analogous definition of conditional mutual information holds through the expected KL-divergence for any three random variables *X*, *Y*, *Z*:

$$𝕀\left(X;Y|Z\right)=𝔼\left[D_{\text{KL}}(p\left(X,Y|Z\right)\|p(X|Z)p(Y|Z))\right].$$

With these definitions in hand, we may define the entropy and conditional entropy for any two random variables *X*, *Y* as

$$ℍ\left(X\right)=𝕀\left(X;X\right)\;ℍ\left(Y|X\right)=ℍ\left(Y\right)-𝕀\left(X;Y\right).$$

This yields the following identities for mutual information and conditional mutual information for any three arbitrary random variables *X*, *Y*, and *Z*:

$$𝕀\left(X;Y\right)=ℍ\left(X\right)-ℍ\left(X|Y\right)=ℍ\left(Y\right)-ℍ\left(Y|X\right),\;𝕀\left(X;Y|Z\right)=ℍ\left(X|Z\right)-ℍ\left(X|Y,Z\right)=ℍ\left(Y|Z\right)-ℍ\left(Y|X,Z\right).$$

Through the chain rule of the KL-divergence and the fact that *D*_KL_(*p* ‖ *p*) = 0 for any probability distribution *p*, we obtain another equivalent definition of mutual information,

$$𝕀\left(X;Y\right)=𝔼\left[D_{\text{KL}}\left(p\left(Y|X\right)\|p\left(Y\right)\right)\right],$$

as well as the chain rule of mutual information:

$$𝕀\left(X;Y_{1},\ldots ,Y_{n}\right)=\sum_{i=1}^{n}𝕀\left(X;Y_{i}|Y_{1},\ldots ,Y_{i-1}\right).$$

Finally, for any three random variables *X*, *Y*, and *Z* which form the Markov chain *X* → *Y* → *Z*, we have the following data-processing inequality:

$$𝕀\left(X;Z\right)\leq 𝕀\left(X;Y\right).$$

Throughout the paper, the random variable *H*_*t*_ will often appear denoting the current history of an agent’s interaction with the environment. We will use *p*_*t*_(*X*) = *p*(*X* | *H*_*t*_) as shorthand notation for the conditional distribution of any random variable *X* given a random realization of an agent’s history *H*_*t*_, at any timestep *t* ∈ [*T*]. Similarly, we denote the entropy and conditional entropy conditioned upon a specific realization of an agent’s history *H*_*t*_, for some timestep *t* ∈ [*T*], as ℍ_*t*_(*X*) ≜ ℍ(*X* | *H*_*t*_ = *H*_*t*_) and ℍ_*t*_(*X* | *Y*) ≜ ℍ_*t*_(*X* | *Y*, *H*_*t*_ = *H*_*t*_), for two arbitrary random variables *X* and *Y*. This notation will also apply analogously to the mutual information 𝕀_*t*_(*X*; *Y*) ≜ 𝕀(*X*; *Y* | *H*_*t*_ = *H*_*t*_) = ℍ_*t*_(*X*) − ℍ_*t*_(*X* | *Y*) = ℍ_*t*_(*Y*) − ℍ_*t*_(*Y* | *X*), as well as the conditional mutual information 𝕀_*t*_(*X*; *Y* | *Z*) ≜ 𝕀(*X*; *Y* | *H*_*t*_ = *H*_*t*_, *Z*), given an arbitrary third random variable, *Z*. A reader should interpret this as recognizing that, while standard information-theoretic quantities average over all associated random variables, an agent attempting to quantify information for the purposes of exploration does so not by averaging over all possible histories that it could potentially experience, but rather by conditioning based on the particular random history *H*_*t*_ that it has currently observed thus far. This dependence on the random realization of history *H*_*t*_ makes all of the aforementioned quantities random variables themselves. The traditional notions of conditional entropy and conditional mutual information given the random variable *H*_*t*_ arise by taking an expectation over histories:

$$\left\{\begin{array}{l} 𝔼\left[{ℍ}_{t}\left(X\right)\right]=ℍ\left(X|H_{t}\right)\\ 𝔼\left[{ℍ}_{t}\left(X|Y\right)\right]=ℍ\left(X|Y,H_{t}\right) \end{array}\right.\;,\;\left\{\begin{array}{l} 𝔼\left[{𝕀}_{t}\left(X;Y\right)\right]=𝕀\left(X;Y|H_{t}\right),\\ 𝔼\left[{𝕀}_{t}\left(X;Y|Z\right)\right]=𝕀\left(X;Y|H_{t},Z\right) \end{array}\right.\;.$$

Additionally, we adopt a similar notation to express a conditional expectation given the random history *H*_*t*_ : 𝔼_*t*_[*X*] ≜ 𝔼[*X* | *H*_*t*_].

## APPENDIX B: EPISODIC REINFORCEMENT LEARNING

In this section, we again specialize the general problem formulation of Continual Learning section, this time by introducing the assumption of episodicity commonly made throughout the reinforcement-learning literature. Thompson Sampling will again reappear as a quintessential algorithm for addressing exploration under an additional assumption that planning across any world model is always computationally feasible. Under this caveat, we survey existing theoretical results which accommodate capacity-limited agents via rate-distortion theory.

### Problem Formulation

We formulate a sequential decision-making problem as an episodic, finite-horizon Markov Decision Process (MDP) (Bellman, 1957; Puterman, 1994) defined by 𝓜 = 〈𝒮, 𝒜, 𝒰, 𝒯, *β*, *H*〉. Here 𝒮 denotes a set of states, 𝒜 is a set of actions, 𝒰 : 𝒮 × 𝒜 → [0, 1] is a deterministic reward or utility function providing evaluative feedback signals, 𝒯 : 𝒮 × 𝒜 → Δ(𝒮) is a transition function prescribing distributions over next states, *β* ∈ Δ(𝒮) is an initial state distribution, and *H* ∈ ℕ is the maximum length or horizon. Within each one of *K* ∈ ℕ episodes, the agent acts for exactly *H* steps beginning with an initial state *s*_1_ ∼ *β*. For each timestep *h* ∈ [*H*], the agent observes the current state *s*_*h*_ ∈ 𝒮, selects action *a*_*h*_ ∼ *π*_*h*_(· | *s*_*h*_) ∈ 𝒜, enjoys a reward *r*_*h*_ = 𝒰(*s*_*h*_, *a*_*h*_) ∈ [0, 1], and transitions to the next state *s*_*h*+1_ ∼ 𝒯( · | *s*_*h*_, *a*_*h*_) ∈ 𝒮.

A stationary, stochastic policy for timestep *h* ∈ [*H*], *π*_*h*_ : 𝒮 → Δ(𝒜), encodes behavior as a mapping from states to distributions over actions. Letting Π ≜ {𝒮 → Δ(𝒜)} denote the class of all stationary, stochastic policies, a non-stationary policy *π* = (*π*_1_, …, *π*_*H*_) ∈ Π^*H*^ is a collection of exactly *H* stationary, stochastic policies whose overall performance in any MDP 𝓜 at timestep *h* ∈ [*H*] when starting at state *s* ∈ 𝒮 and taking action *a* ∈ 𝒜 is assessed by its associated action-value function $Q_{𝓜,h}^{\pi }\left(s,a\right)=𝔼\left[\sum_{h\prime =h}^{H}𝒰\left(s_{h\prime },a_{h\prime }\right)|s_{h}=s,a_{h}=a\right]$, where the expectation integrates over randomness in the action selections and transition dynamics. Taking the corresponding value function as $V_{𝓜,h}^{\pi }\left(s\right)={𝔼}_{a∼\pi_{h}\left(\cdot |s\right)}\left[Q_{𝓜,h}^{\pi }\left(s,a\right)\right]$, we define the optimal policy *π*^⋆^ = $\left(\pi_{1}^{⋆},\pi_{2}^{⋆},\ldots ,\pi_{H}^{⋆}\right)$ as achieving supremal value $V_{𝓜,h}^{⋆}\left(s\right)=\underset{\pi \in \Pi^{H}}{\sup}V_{𝓜,h}^{\pi }\left(s\right)$ for all *s* ∈ 𝒮, *h* ∈ [*H*].

We let *τ*_*k*_ = $\left(s_{1}^{\left(k\right)},a_{1}^{\left(k\right)},r_{1}^{\left(k\right)},\ldots ,s_{H}^{\left(k\right)},a_{H}^{\left(k\right)},r_{H}^{\left(k\right)},s_{H+1}^{\left(k\right)}\right)$ be the random variable denoting the trajectory experienced by the agent in the *k*th episode. Meanwhile, *H*_*k*_ = {*τ*_1_, *τ*_2_, …, *τ*_*k*−1_} ∈ 𝓗_*k*_ is the random variable representing the entire history of the agent’s interaction within the environment at the start of the *k*th episode. As is standard in Bayesian reinforcement learning (Bellman & Kalaba, 1959; Duff, 2002; Ghavamzadeh et al., 2015), neither the transition function nor the reward function are known to the agent and, consequently, both are treated as random variables.

Just as in the previous section but with a slight abuse of notation, we will use *p*_*k*_(*X*) = *p*(*X* | *H*_*k*_) as shorthand notation for the conditional distribution of any random variable *X* given a random realization of an agent’s history *H*_*k*_ ∈ 𝓗, at any episode *k* ∈ [*K*]. Furthermore, we will denote the entropy and conditional entropy conditioned upon a specific realization of an agent’s history *H*_*k*_, for some episode *k* ∈ [*K*], as ℍ_*k*_(*X*) ≜ ℍ(*X* | *H*_*k*_ = *H*_*k*_) and ℍ_*k*_(*X* | *Y*) ≜ ℍ_*k*_(*X* | *Y*, *H*_*k*_ = *H*_*k*_), for two arbitrary random variables *X* and *Y*. This notation will also apply analogously to mutual information: 𝕀_*k*_(*X*; *Y*) ≜ 𝕀(*X*; *Y* | *H*_*k*_ = *H*_*k*_) = ℍ_*k*_(*X*) − ℍ_*k*_(*X* | *Y*) = ℍ_*k*_(*Y*) − ℍ_*k*_(*Y* | *X*). We reiterate that a reader should interpret this as recognizing that, while standard information-theoretic quantities average over all associated random variables, an agent attempting to quantify information for the purposes of exploration does so not by averaging over all possible histories that it could potentially experience, but rather by conditioning based on the particular random history *H*_*k*_. The dependence on the realization of a random history *H*_*k*_ makes 𝕀_*k*_(*X*; *Y*) a random variable and the usual conditional mutual information arises by integrating over this randomness: 𝔼[𝕀_*k*_(*X*; *Y*)] = 𝕀(*X*; *Y* | *H*_*k*_). Additionally, we will also adopt a similar notation to express a conditional expectation given the random history *H*_*k*_ : 𝔼_*k*_[*X*] ≜ 𝔼[*X* | *H*_*k*_].

### Posterior Sampling for Reinforcement Learning

A natural starting point for addressing the exploration challenge in a principled manner is via Thompson Sampling (Russo et al., 2018; Thompson, 1933). The Posterior Sampling for Reinforcement Learning (PSRL) (Abbasi-Yadkori & Szepesvari, 2014; Agrawal & Jia, 2017; Lu & Van Roy, 2019; Osband et al., 2013; Osband & Van Roy, 2014, 2017; Strens, 2000) algorithm (given as Algorithm 6) does this by, in each episode *k* ∈ [*K*], sampling a candidate MDP 𝓜_*k*_ ∼ *p*_*k*_(𝓜^⋆^) and executing its optimal policy in the environment *π*^(*k*)^ = $\pi_{{𝓜}_{k}}^{⋆}$; notably, such posterior sampling guarantees the hallmark probability-matching principle of Thompson Sampling: *p*_*k*_(𝓜_*k*_ = *M*) = *p*_*k*_(𝓜^⋆^ = *M*), ∀*M* ∈ 𝔐, *k* ∈ [*K*]. The resulting trajectory *τ*_*k*_ leads to a new history *H*_*k*+1_ = *H*_*k*_ ∪ *τ*_*k*_ and an updated posterior over the true MDP *p*_*k*+1_(𝓜^⋆^).

Unfortunately, for complex environments, pursuit of the exact MDP 𝓜^⋆^ may be an entirely infeasible goal, akin to pursuing an optimal action *A*^⋆^ within a multi-armed bandit problem. A MDP representing control of a real-world, physical system, for example, suggests that learning the associated transition function requires the agent internalize laws of physics and motion with near-perfect accuracy. More formally, identifying 𝓜^⋆^ demands the agent obtain exactly ℍ_1_(𝓜^⋆^) bits of information from the environment which, under an uninformative prior, may either be prohibitively large by far exceeding the agent’s capacity constraints or be simply impractical under time and resource constraints (Lu et al., 2023).

### Rate-Distortion Theory for Target MDPs

To remedy the intractabilities imposed by PSRL when an agent must contend with an overwhelmingly-complex environment, we once again turn to rate-distortion theory as a tool for defining an information-theoretic surrogate than an agent may use to prioritize its information acquisition strategy in lieu of 𝓜^⋆^. If one were to follow the rate-distortion optimization of Equation 2, this would suggest identifying a channel *δ*_*t*_(*π*_*χ*_ | 𝓜^⋆^) that directly maps a bounded agent’s beliefs about 𝓜^⋆^ to a target policy *π*_*χ*_. For the purposes of analysis, Arumugam and Van Roy (2022) instead perform lossy MDP compression with the interpretation that various facets of the true MDP 𝓜^⋆^ must be discarded by a capacity-limited agent who can only hope identify a simplified world model that strives to retain as many salient details as possible. Implicit to such an approach is an assumption that the act of planning (that is, mapping any MDP *M* ∈ 𝔐 to its optimal policy $\pi_{M}^{⋆}$) can always be done in a computationally-efficient manner irrespective of the agent’s capacity limitations. From a mechanistic perspective, this is likely implausible for both artificial agents in large-scale, high-dimensional environments of interest as well as biological agents (Ho et al., 2022). On the other hand, this construction induces a Markov chain 𝓜^⋆^ − $\tilde{𝓜}$ − *π*_*χ*_, where $\tilde{𝓜}$ denotes the compressed world model; by the data-processing inequality, we have for all *k* ∈ [*K*] that 𝕀_*k*_(𝓜^⋆^; *π*_*χ*_) ≤ 𝕀_*k*_(𝓜^⋆^; $\tilde{𝓜}$), such that minimizing the rate of the lossy MDP compression must also limit the amount of information that flows from the agent’s beliefs about the world to the executed behavior policy.

For the precise details of this MDP compression, we first require (just as with any lossy compression problem) the specification of an information source to be compressed as well as a distortion function that quantifies the loss of fidelity between uncompressed and compressed values. Akin to the multi-armed bandit setting, we will take the agent’s current beliefs *p*_*k*_(𝓜^⋆^) as the information source to be compressed in each episode. Unlike in the bandit setting, however, the choice of distortion function *d* : 𝔐 × 𝔐 → ℝ_≥0_ presents an opportunity for the agent designer to be judicious in specifying which aspects of the environment are preserved in the agent’s compressed view of the world. From a biological perspective, one might hypothesize that some combination of nature and evolutionary pressures adapt suitable distortion functions for biological decision-making agents.

It is fairly well accepted that human beings do not model all facets of the environment when making decisions (Gigerenzer & Goldstein, 1996; Simon, 1956) and the choice of which details are deemed salient enough to warrant retention in the mind of an agent is precisely governed by the choice of distortion function. In the computational reinforcement-learning literature, this reality has called into question longstanding approaches to model-based reinforcement learning (Littman, 2015; Sutton, 1991; Sutton & Barto, 1998) which use standard maximum-likelihood estimation techniques that endeavor to learn the exact model (𝒰, 𝒯) that governs the underlying MDP. The end result has been a flurry of recent work (Abachi et al., 2020; Asadi et al., 2018; Ayoub et al., 2020; Cui et al., 2020; D’Oro et al., 2020; Farahmand, 2018; Farahmand et al., 2017; Grimm et al., 2020, 2021, 2022; Nair et al., 2020; Nikishin et al., 2022; Oh et al., 2017; Schrittwieser et al., 2020; Silver et al., 2017; Voelcker et al., 2022) which eschews the traditional maximum-likelihood objective in favor of various surrogate objectives which restrict the focus of the agent’s modeling towards specific aspects of the environment. As the core goal of endowing a decision-making agent with its own internal model of the world is to facilitate model-based planning (Bertsekas, 1995), central among these recent approaches is the value-equivalence principle (Grimm et al., 2020, 2021, 2022) which provides mathematical clarity on how surrogate models can still enable lossless planning relative to the true model of the environment.

For any arbitrary MDP 𝓜 with model (𝒰, 𝒯) and any stationary, stochastic policy *π* : 𝒮 → Δ(𝒜), define the Bellman operator ${𝓑}_{𝓜}^{\pi }$ : {𝒮 → ℝ} → {𝒮 → ℝ} as follows:

$${𝓑}_{𝓜}^{\pi }V\left(s\right)≜{𝔼}_{a∼\pi \left(\cdot |s\right)}\left[𝒰\left(s,a\right)+{𝔼}_{s\prime ∼𝒯\left(\cdot |s,a\right)}\left[V\left(s^{\prime }\right)\right]\right].$$

The Bellman operator is a foundational tool in dynamic-programming approaches to reinforcement learning (Bertsekas, 1995) and gives rise to the classic Bellman equation: for any MDP 𝓜 = 〈𝒮, 𝒜, 𝒰, 𝒯, *β*, *H*〉 and any non-stationary policy *π* = (*π*_1_, …, *π*_*H*_), the value functions induced by *π* satisfy $V_{𝓜,h}^{\pi }\left(s\right)={𝓑}_{𝓜}^{\pi_{h}}V_{𝓜,h+1}^{\pi }\left(s\right)$, for all *h* ∈ [*H*] and with $V_{𝓜,H+1}^{\pi }\left(s\right)$ = 0, ∀*s* ∈ 𝒮. For any two MDPs 𝓜 = 〈𝒮, 𝒜, 𝒰, 𝒯, *β*, *H*〉 and $\hat{𝓜}$ = 〈𝒮, 𝒜, $\hat{𝒰}$, $\hat{𝒯}$, *β*, *H*〉, Grimm et al. (2020) define a notion of equivalence between them despite their differing models. For any policy class Π ⊆ {𝒮 → Δ(𝒜)} and value function class 𝒱 ⊆ {𝒮 → ℝ}, 𝓜 and $\hat{𝓜}$ are value equivalent with respect to Π and 𝒱 if and only if ${𝓑}_{𝓜}^{\pi }V={𝓑}_{\hat{𝓜}}^{\pi }V$, ∀*π* ∈ Π, *V* ∈ 𝒱. In words, two different models are deemed value equivalent if they induce identical Bellman updates under any pair of policy and value function from Π × 𝒱. Grimm et al. (2020) prove that when Π = {𝒮 → Δ(𝒜)} and 𝒱 = {𝒮 → ℝ}, the set of all exactly value-equivalent models is a singleton set containing only the true model of the environment. By recognizing that the ability to plan over all arbitrary behaviors is not necessarily in the agent’s best interest and restricting focus to decreasing subsets of policies Π ⊂ {𝒮 → Δ(𝒜)} and value functions 𝒱 ⊂ {𝒮 → ℝ}, the space of exactly value-equivalent models is monotonically increasing.

Still, however, exact value equivalence still presumes that an agent has the capacity for planning with complete fidelity to the true environment; more plausibly, an agent may only have the resources to plan in an approximately-value-equivalent manner (Grimm et al., 2022). For brevity, let ℜ ≜ {𝒮 × 𝒜 → [0, 1]} and 𝔗 ≜ {𝒮 × 𝒜 → Δ(𝒮)} denote the classes of all reward functions and transition functions, respectively. Recall that, with 〈𝒮, 𝒜, *β*, *H*〉 all known, the uncertainty in a random MDP 𝓜 is entirely driven by its model (𝓡, 𝒯) such that we may think of the support of 𝓜^⋆^ as supp(𝓜^⋆^) = 𝔐 ≜ ℜ × 𝔗. We define a distortion function on pairs of MDPs *d* : 𝔐 × 𝔐 → ℝ_≥0_ for any Π ⊆ {𝒮 → Δ(𝒜)}, 𝒱 ⊆ {𝒮 → ℝ} as

$$d_{\Pi ,𝒱}\left(𝓜,\hat{𝓜}\right)=\underset{\begin{array}{c} \pi \in \Pi \\ V\in 𝒱 \end{array}}{\text{sup}}{\left\|{𝓑}_{𝓜}^{\pi }V-{𝓑}_{\hat{𝓜}}^{\pi }V\right\|}_{\infty }^{2}=\underset{\begin{array}{c} \pi \in \Pi \\ V\in 𝒱 \end{array}}{\text{sup}}{\left(\underset{s\in 𝒮}{\text{sup}}|{𝓑}_{𝓜}^{\pi }V\left(s\right)-{𝓑}_{\hat{𝓜}}^{\pi }V\left(s\right)\right)}^{2}.$$

In words, *d*_Π,𝒱_ is the supremal squared Bellman error between MDPs 𝓜 and $\hat{𝓜}$ across all states *s* ∈ 𝒮 with respect to the policy class Π and value function class 𝒱. With an information source and distortion function defined, Arumugam and Van Roy (2022) employ the following rate-distortion function that articulates the lossy MDP compression a capacity-limited decision agent performs to identify a simplified MDP to pursue instead of 𝓜^⋆^:

$${𝓡}_{k}\left(D\right)=\underset{p\left(\tilde{𝓜}|{𝓜}^{⋆}\right)}{\text{inf}}{𝕀}_{k}\left({𝓜}^{⋆};\tilde{𝓜}\right)\;\text{such}\;\text{that}\;{𝔼}_{k}\left[d\left({𝓜}^{⋆},\tilde{𝓜}\right)\right]\leq D.$$ (5)

By definition, the target MDP $\tilde{𝓜}$_*k*_ that achieves this rate-distortion limit will demand that the agent acquire fewer bits of information than what is needed to identify 𝓜^⋆^. Once again, by virtue of Fact 1, this claim is guaranteed for all *k* ∈ [*K*] and any *D* > 0: 𝓡_*k*_(*D*) ≤ 𝓡_*k*_(0) ≤ 𝕀_*k*_(𝓜^⋆^; 𝓜^⋆^) = ℍ_*k*_(𝓜^⋆^). Crucially, however, the use of the value-equivalence principle in the distortion function ensures that agent capacity is allocated towards preserving the regions of the world model needed to plan over behaviors as defined through Π, 𝒱. Arumugam and Van Roy (2022) establish an information-theoretic Bayesian regret bound for a posterior-sampling algorithm (given as Algorithm 7) that performs probability matching with respect to $\tilde{𝓜}$_*k*_ in each episode *k* ∈ [*K*], instead of 𝓜^⋆^.

Just as with the BLASTS algorithm for the multi-armed bandit setting, this VSRL algorithm directly couples an agent’s exploratory choices in each episode to the epistemic uncertainty it maintains over the resource-rational learning target $\tilde{𝓜}$_*k*_ which it aspires to learn. The bound communicates that an agent with limited capacity must tolerate a higher distortion threshold *D* and pursue the resulting compressed MDP that bears less fidelity to the original MDP; in exchange, the resulting number of bits needed from the environment to identify such a simplified model of the world is given as 𝓡_1_(*D*) and guaranteed to be less than the entropy of 𝓜^⋆^. Additionally, just as with the regret bound for BLASTS, one can express a near-identical result through the associated distortion-rate function. In particular, this encourages a particular notion of agent capacity as a limit *R* ∈ ℝ_≥0_ on the number of bits an agent may obtain from its interactions with the environment. Subject to this constraint, the fundamental limit on the amount of expected distortion incurred is given by

$${𝒟}_{t}\left(R\right)=\underset{p\left(\tilde{𝓜}|{𝓜}^{⋆}\right)}{\text{inf}}{𝔼}_{k}\left[d\left({𝓜}^{⋆},\tilde{𝓜}\right)\right]\;\text{such}\;\text{that}\;{𝕀}_{k}\left({𝓜}^{⋆};\tilde{𝓜}\right)\leq R.$$ (6)

Embracing this distortion-rate function and taking the VSRL distortion threshold as *D* = 𝒟_1_(*R*) allows for a performance guarantee that explicitly accounts for the agent capacity limits.

In summary, under a technical assumption of episodicity for the purposes of analysis, the theoretical results surveyed in this section parallel those for multi-armed bandits. While computational experiments for this episodic reinforcement learning setting have not yet been established due to the computational efficiency of running the Blahut-Arimoto algorithm for such a lossy MDP compression problem, the core takeaway of this section is that there is strong theoretical justification for using these tools from rate-distortion theory to empirically study capacity-limited sequential decision-making agents. We refer readers to the discussion in Appendix B.3 of Arumugam and Van Roy (2022) for consideration of how these ideas might productively scale with deep reinforcement learning to high-dimensional environments that necessitate the use of function approximation.

## APPENDIX C: REGRET ANALYSIS FOR RATE-DISTORTION THOMPSON SAMPLING

Recall the multi-armed bandit problem formulation of Multi-Armed Bandit section. For a fixed choice of environment 𝓔, the performance of an agent is assessed through the regret of its policies over *T* time periods

$$\text{Regret}\left({\left\{\pi_{t}\right\}}_{t\in \left[T\right]},𝓔\right)=𝔼\left[\sum_{t=1}^{T}\left(\bar{\rho }\left(A^{⋆}\right)-\bar{\rho }\left(A_{t}\right)\right)|𝓔\right].$$

Since the environment is itself a random quantity, we integrate over this randomness with respect to the prior *η*_1_(𝓔) to arrive at the Bayesian regret:

$$\text{BayesRegret}\left({\left\{\pi_{t}\right\}}_{t\in \left[T\right]}\right)=𝔼\left[\text{Regret}({\left\{\pi_{t}\right\}}_{t\in \left[T\right]},𝓔)\right]=𝔼\left[\sum_{t=1}^{T}\left(\bar{\rho }\left(A^{⋆}\right)-\bar{\rho }\left(A_{t}\right)\right)\right].$$

The customary goal within a multi-armed bandit problem is to identify an optimal action *A*^⋆^ and provably-efficient bandit learning emerges from algorithms whose Bayesian regret can be bounded from above. We aim to prove the following upper bound for the Bayesian regret of Rate-Distortion Thompson Sampling (Algorithm 5).

**Theorem 1.** *For any* *D* ≥ 0,

$$\text{BayesRegret}\left({\left\{\pi_{t}^{\text{RDTS}}\right\}}_{t\in \left[T\right]}\right)\leq \sqrt{\frac{1}{2}|𝒜|T{𝓡}_{1}\left(D\right)}+T\sqrt{D}.$$

When *D* = 0 and the agent designer is not willing to tolerate any sub-optimality relative to *A*^⋆^, Fact 1 allows this bound to recover the guarantee of TS exactly. At the other extreme, increasing *D* to 1 (recall that mean reward are bounded in [0, 1]) allows 𝓡_1_(*D*) = 0 and the agent has nothing to learn from the environment but also suffers the linear regret of *T*. Naturally, the “sweet spot” is to entertain intermediate values of *D* where smaller values will lead to larger amounts of information 𝓡_1_(*D*) needed to identify the corresponding target action, but not as many bits as what learning *A*^⋆^ necessarily entails.

It may often be sensible to also consider a scenario where an agent designer is unable to precisely specify a reasonable threshold on expected distortion *D* and can, instead, only characterize a limit on the amount of information an agent may acquire from the environment *R* > 0. One might interpret this as a notion of capacity which differs quite fundamentally from other notions examined in prior work (Gershman, 2023; Lai & Gershman, 2021) (see Discussion section for a more in-depth comparison). For this, we may consider the distortion-rate function

$${𝒟}_{t}\left(R\right)=\underset{p\left(\tilde{𝒜}|𝓔\right)}{\text{inf}}{𝔼}_{t}\left[d\left(\tilde{𝒜},𝓔\right)\right]\;\text{such}\;\text{that}\;{𝕀}_{t}\left(𝓔;\tilde{𝒜}\right)\leq R,$$ (7)

which quantifies the fundamental limit of lossy compression subject to a rate constraint, rather than the distortion threshold of 𝓡(*D*). Similar to the rate-distortion function, however, the distortion rate function also adheres to the three properties outlined in Fact 1. More importantly, it is the inverse of the rate-distortion function such that 𝓡_*t*_(𝒟_*t*_(*R*)) = *R* for any *t* ∈ [*T*] and *R* > 0. Consequently, by selecting *D* = 𝒟_1_(*R*) as input to Algorithm 5, we immediately recover the following corollary to Theorem 1 that provides an information-theoretic Bayesian regret bound in terms of agent capacity, rather than a threshold on expected distortion.

**Corollary 1.** *For any* *R* > 0,

$$\text{BayesRegret}\left({\left\{\pi_{t}^{\text{RDTS}}\right\}}_{t\in \left[T\right]}\right)\leq \sqrt{\frac{1}{2}|𝒜|TR}+T\sqrt{{𝒟}_{1}\left(R\right)}.$$

The semantics of this performance guarantee are identical to those of Theorem 1, only now expressed explicitly through the agent’s capacity *R*. Namely, when the agent has no capacity for learning *R* = 0, *D*_1_(*R*) = 1 and the agent incurs linear regret of *T*. Conversely, with sufficient capacity *R* = ℍ_1_(*A*^⋆^), *D*_1_(*R*) = 0 and we recover the regret bound of Thompson Sampling. Intermediate values of agent capacity will result in an agent that fully utilizes its capacity to acquire no more than *R* bits of information from the environment, resulting in the minimum possible expected distortion quantified by 𝒟_1_(*R*).

We begin our analysis by establishing the following fact, which also appears in the proof of Lemma 3 of Arumugam and Van Roy (2021a):

**Fact 2.** *For any target action* $\tilde{A}$ *and any time period t* ∈ [*T*],

$${𝕀}_{t}\left(\tilde{A};\left(A_{t},O_{t+1}\right)\right)={𝕀}_{t}\left(𝓔;\tilde{A}\right)-{𝕀}_{t}\left(𝓔;\tilde{A}|A_{t},O_{t+1}\right).$$

*Proof.* Recall that for any *t* ∈ [*T*], *H*_*t*+1_ = (*H*_*t*_, *A*_*t*_, *O*_*t*+1_). Moreover, no action-observation pair can offer more information about any target action $\tilde{A}$ than the environment 𝓔 itself. Thus, we have that ∀*t* ∈ [*T*], *H*_*t*_ ⊥ $\tilde{A}$ | 𝓔, which implies 𝕀_*t*_($\tilde{A}$; (*A*_*t*_, *O*_*t*+1_) | 𝓔) = 0. By the chain rule of mutual information,

$${𝕀}_{t}\left(𝓔;\tilde{A}\right)={𝕀}_{t}\left(𝓔;\tilde{A}\right)+{𝕀}_{t}\left(\tilde{A};\left(A_{t},O_{t+1}\right)|𝓔\right)={𝕀}_{t}\left(𝓔,\left(A_{t},O_{t+1}\right);\tilde{A}\right).$$

Applying the chain rule of mutual information a second time yields

$${𝕀}_{t}\left(𝓔;\tilde{A}\right)={𝕀}_{t}\left(𝓔,\left(A_{t},O_{t+1}\right);\tilde{A}\right)={𝕀}_{t}\left(\tilde{A};\left(A_{t},O_{t+1}\right)\right)+{𝕀}_{t}\left(𝓔;\tilde{A}|A_{t},O_{t+1}\right).$$

Finally, simply re-arranging terms gives

$${𝕀}_{t}\left(\tilde{A};\left(A_{t},O_{t+1}\right)\right)={𝕀}_{t}\left(𝓔;\tilde{A}\right)-{𝕀}_{t}\left(𝓔;\tilde{A}|A_{t},O_{t+1}\right),$$

as desired.

**Lemma 1.** *For any D* > 0 *and all t* ∈ [*T*],

$${𝔼}_{t}\left[{𝓡}_{t+1}\left(D\right)\right]\leq {𝓡}_{t}\left(D\right)-{𝕀}_{t}\left({\tilde{A}}_{t};\left(A_{t},O_{t+1}\right)\right).$$

*Proof.* By definition, $\tilde{A}$_*t*_ achieves the rate-distortion limit such that 𝔼_*t*_[*d*($\tilde{A}$_*t*_, 𝓔)] ≤ *D*. Recall that, by Fact 1, the rate-distortion function is a non-increasing function in its argument. This implies that for any *D*_1_ ≤ *D*_2_, 𝓡_*t*+1_(*D*_2_) ≤ 𝓡_*t*+1_(*D*_1_). Applying this fact to the inequality above and taking expectations, we obtain

$${𝔼}_{t}\left[{𝓡}_{t+1}\left(D\right)\right]\leq {𝔼}_{t}\left[{𝓡}_{t+1}\left({𝔼}_{t}\left[d\left({\tilde{A}}_{t},𝓔\right)\right]\right)\right].$$

Observe by the tower property of expectation that

$${𝔼}_{t}\left[{𝓡}_{t+1}\left(D\right)\right]\leq {𝔼}_{t}\left[{𝓡}_{t+1}\left({𝔼}_{t}\left[d\left({\tilde{A}}_{t},𝓔\right)\right]\right)\right]={𝔼}_{t}\left[{𝓡}_{t+1}\left({𝔼}_{t}\left[{𝔼}_{t+1}\left[d\left({\tilde{A}}_{t},𝓔\right)\right]\right]\right)\right].$$

Moreover, from Fact 1, we recall that the rate-distortion function is a convex function. Consequently, by Jensen’s inequality, we have

$${𝔼}_{t}\left[{𝓡}_{t+1}\left(D\right)\right]\leq {𝔼}_{t}\left[{𝓡}_{t+1}\left({𝔼}_{t}\left[d\left({\tilde{A}}_{t},𝓔\right)\right]\right)\right]={𝔼}_{t}\left[{𝓡}_{t+1}\left({𝔼}_{t}\left[{𝔼}_{t+1}\left[d\left({\tilde{A}}_{t},𝓔\right)\right]\right]\right)\right]\leq {𝔼}_{t}\left[{𝓡}_{t+1}\left({𝔼}_{t+1}\left[d\left({\tilde{A}}_{t},𝓔\right)\right]\right)\right].$$

Inspecting the definition of the rate-distortion in the expectation, we see that

$${𝓡}_{t+1}\left(D\right)=\underset{p\left(\tilde{A}|𝓔\right)}{\text{inf}}{𝕀}_{t+1}\left(𝓔;\tilde{A}\right)\;\text{such}\;\text{that}\;{𝔼}_{t+1}\left[d\left(\tilde{A},𝓔\right)\right]\leq D,$$

which immediately implies

$${𝓡}_{t+1}\left({𝔼}_{t+1}\left[d\left({\tilde{A}}_{t},𝓔\right)\right]\right)\leq {𝕀}_{t+1}\left(𝓔;{\tilde{A}}_{t}\right).$$

Substituting back into the earlier expression, we have

$${𝔼}_{t}\left[{𝓡}_{t+1}\left(D\right)\right]\leq {𝔼}_{t}\left[{𝕀}_{t+1}\left(𝓔;{\tilde{A}}_{t}\right)\right]={𝔼}_{t}\left[{𝕀}_{t}\left(𝓔;{\tilde{A}}_{t}|A_{t},O_{t+1}\right)\right]={𝕀}_{t}\left(𝓔;{\tilde{A}}_{t}|A_{t},O_{t+1}\right).$$

We now apply Fact 2 to arrive at

$${𝔼}_{t}\left[{𝓡}_{t+1}\left(D\right)\right]\leq {𝕀}_{t}\left(𝓔;{\tilde{A}}_{t}|A_{t},O_{t+1}\right)={𝕀}_{t}\left(𝓔;{\tilde{A}}_{t}\right)-{𝕀}_{t}\left({\tilde{A}}_{t};\left(A_{t},O_{t+1}\right)\right).$$

Since, by definition, $\tilde{A}$_*t*_ achieves the rate-distortion limit at time period *t*, we know that 𝕀_*t*_(𝓔; $\tilde{A}$_*t*_) = 𝓡_*t*_(*D*). Applying this fact yields the desired inequality:

$${𝔼}_{t}\left[{𝓡}_{t+1}\left(D\right)\right]\leq {𝕀}_{t}\left(𝓔;{\tilde{A}}_{t}\right)-{𝕀}_{t}\left({\tilde{A}}_{t};\left(A_{t},O_{t+1}\right)\right)={𝓡}_{t}\left(D\right)-{𝕀}_{t}\left({\tilde{A}}_{t};\left(A_{t},O_{t+1}\right)\right).$$

Lemma 1 shows that the expected amount of information needed from the environment in each successive time period is non-increasing and further highlights two possible sources for this improvement: (1) a change in learning target from $\tilde{A}$_*t*_ to $\tilde{A}$_*t*+1_ and (2) information acquired about $\tilde{A}$_*t*_ in the current time period, 𝕀_*t*_($\tilde{A}$_*t*_; (*A*_*t*_, *O*_*t*+1_)). With this in hand, we can obtain control over the cumulative information gain of an agent across all time periods using the learning target identified under our prior, following an identical argument as Arumugam and Van Roy (2022).

**Lemma 2.** *For any fixed D* > 0 *and any t* ∈ [*T*],

$${𝔼}_{t}\left[\sum_{t\prime =t}^{T}{𝕀}_{t\prime }({\tilde{A}}_{t\prime };\left(A_{t\prime },O_{t\prime +1}\right))\right]\leq {𝓡}_{t}\left(D\right).$$

*Proof.* Observe that we can apply Lemma 1 directly to each term of the sum and obtain

$${𝔼}_{t}\left[\sum_{t\prime =t}^{T}{𝕀}_{t\prime }({\tilde{A}}_{t\prime };\left(A_{t\prime },O_{t\prime +1}\right))\right]\leq {𝔼}_{t}\left[\sum_{t\prime =t}^{T}\left({𝓡}_{t\prime }\left(D\right)-{𝔼}_{t\prime }\left[{𝓡}_{t\prime +1}\left(D\right)\right]\right)\right].$$

Applying linearity of expectation and breaking apart the sum, we have

$$\begin{array}{l} {𝔼}_{t}\left[\sum_{t\prime =t}^{T}{𝕀}_{t\prime }({\tilde{A}}_{t\prime };\left(A_{t\prime },O_{t\prime +1}\right))\right]\leq {𝔼}_{t}\left[\sum_{t\prime =t}^{T}\left({𝓡}_{t\prime }\left(D\right)-{𝔼}_{t\prime }\left[{𝓡}_{t\prime +1}\left(D\right)\right]\right)\right]\\ \;=\sum_{t\prime =t}^{T}{𝔼}_{t}\left[{𝓡}_{t\prime }\left(D\right)\right]-\sum_{t\prime =t}^{T}{𝔼}_{t}\left[{𝔼}_{t\prime }\left[{𝓡}_{t\prime +1}\left(D\right)\right]\right]\\ \;\leq \sum_{t\prime =t}^{T}{𝔼}_{t}\left[{𝓡}_{t\prime }\left(D\right)\right]-\sum_{t\prime =t}^{T-1}{𝔼}_{t}\left[{𝔼}_{t\prime }\left[{𝓡}_{t\prime +1}\left(D\right)\right]\right]\\ \;={𝔼}_{t}\left[{𝓡}_{t}\left(D\right)\right]+\sum_{t\prime =t+1}^{T}{𝔼}_{t}\left[{𝓡}_{t\prime }\left(D\right)\right]-\sum_{t\prime =t}^{T-1}{𝔼}_{t}\left[{𝔼}_{t\prime }\left[{𝓡}_{t\prime +1}\left(D\right)\right]\right]\\ \;={𝓡}_{t}\left(D\right)+\sum_{t\prime =t+1}^{T}{𝔼}_{t}\left[{𝓡}_{t\prime }\left(D\right)\right]-\sum_{t\prime =t}^{T-1}{𝔼}_{t}\left[{𝔼}_{t\prime }\left[{𝓡}_{t\prime +1}\left(D\right)\right]\right]. \end{array}$$

We may complete the proof by applying the tower property of expectation and then re-indexing the last summation

$$\begin{array}{l} {𝔼}_{t}\left[\sum_{t\prime =t}^{T}{𝕀}_{t\prime }({\tilde{A}}_{t\prime };\left(A_{t\prime },O_{t\prime +1}\right))\right]\leq {𝓡}_{t}\left(D\right)+\sum_{t\prime =t+1}^{T}{𝔼}_{t}\left[{𝓡}_{t\prime }\left(D\right)\right]-\sum_{t\prime =t}^{T-1}{𝔼}_{t}\left[{𝔼}_{t\prime }\left[{𝓡}_{t\prime +1}\left(D\right)\right]\right]\\ \;={𝓡}_{t}\left(D\right)+\sum_{t\prime =t+1}^{T}{𝔼}_{t}\left[{𝓡}_{t\prime }\left(D\right)\right]-\sum_{t\prime =t}^{T-1}{𝔼}_{t}\left[{𝓡}_{t\prime +1}\left(D\right)\right]\\ \;={𝓡}_{t}\left(D\right)+\sum_{t\prime =t+1}^{T}{𝔼}_{t}\left[{𝓡}_{t\prime }\left(D\right)\right]-\sum_{t\prime =t+1}^{T}{𝔼}_{t}\left[{𝓡}_{t\prime }\left(D\right)\right]\\ \;={𝓡}_{t}\left(D\right). \end{array}$$

With all of these tools in hand, we may now establish an information-theoretic regret bound. For each time period *t* ∈ [*T*], define the information ratio as

$$\Gamma_{t}≜\frac{{𝔼}_{t}{\left[\bar{\rho }\left({\tilde{A}}_{t}\right)-\bar{\rho }\left(A_{t}\right)\right]}^{2}}{{𝕀}_{t}\left({\tilde{A}}_{t};\left(A_{t},O_{t+1}\right)\right)}.$$

Intuitively, the information ratio is a “conversation factor” that converts bits of information an agent acquires from interacting with the environment at a given time period into units of squared regret.

**Theorem 2.** For any *D* > 0, *if* ∀*t* ∈ [*T*] Γ_*t*_ ≤ $\bar{\Gamma }$ < ∞, *then*

$$𝔼\left[\sum_{t=1}^{T}\left(\bar{\rho }\left(A^{⋆}\right)-\bar{\rho }\left(A_{t}\right)\right)\right]\leq \sqrt{\bar{\Gamma }T{𝓡}_{1}\left(D\right)}+T\sqrt{D}.$$

*Proof.* First, we establish a simple regret decomposition

$$𝔼\left[\sum_{t=1}^{T}\left(\bar{\rho }\left(A^{⋆}\right)-\bar{\rho }\left(A_{t}\right)\right)\right]=𝔼\left[\sum_{t=1}^{T}\left(\bar{\rho }\left(A^{⋆}\right)-\bar{\rho }\left({\tilde{A}}_{t}\right)+\bar{\rho }\left({\tilde{A}}_{t}\right)-\bar{\rho }\left(A_{t}\right)\right)\right]=𝔼\left[\sum_{t=1}^{T}\left(\bar{\rho }\left(A^{⋆}\right)-\bar{\rho }\left({\tilde{A}}_{t}\right)\right)\right]+𝔼\left[\sum_{t=1}^{T}\left(\bar{\rho }\left({\tilde{A}}_{t}\right)-\bar{\rho }\left(A_{t}\right)\right)\right],$$

where the first term captures our cumulative performance shortfall by pursuing a learning target $\tilde{A}$_*t*_ in each time period, rather than *A*^⋆^, while the second term captures our regret with respect to each target. The latter term is also known as the satisficing regret (Russo & Van Roy, 2022). Focusing on the first term, we may apply the tower property of expectation to leverage the fact that each target action $\tilde{A}$_*t*_ achieves the rate-distortion limit and, therefore, has bounded expected distortion:

$$\begin{array}{l} 𝔼\left[\sum_{t=1}^{T}\left(\bar{\rho }\left(A^{⋆}\right)-\bar{\rho }\left({\tilde{A}}_{t}\right)\right)\right]=𝔼\left[\sum_{t=1}^{T}{𝔼}_{t}\left[\bar{\rho }\left(A^{⋆}\right)-\bar{\rho }\left({\tilde{A}}_{t}\right)\right]\right]\\ \;=𝔼\left[\sum_{t=1}^{T}{𝔼}_{t}\left[\left|\bar{\rho }\left(A^{⋆}\right)-\bar{\rho }\left({\tilde{A}}_{t}\right)\right|\right]\right]\\ \;=𝔼\left[\sum_{t=1}^{T}{𝔼}_{t}\left[\sqrt{{\left(\bar{\rho }\left(A^{⋆}\right)-\bar{\rho }\left({\tilde{A}}_{t}\right)\right)}^{2}}\right]\right]\\ \;\leq 𝔼\left[\sum_{t=1}^{T}\sqrt{{𝔼}_{t}\left[{\left(\bar{\rho }\left(A^{⋆}\right)-\bar{\rho }\left({\tilde{A}}_{t}\right)\right)}^{2}\right]}\right]\\ \;=𝔼\left[\sum_{t=1}^{T}\sqrt{{𝔼}_{t}\left[d\left({\tilde{A}}_{t},𝓔\right)\right]}\right]\\ \;\leq 𝔼\left[\sum_{t=1}^{T}\sqrt{D}\right]\\ \;=T\sqrt{D}, \end{array}$$

where the first inequality is due to Jensen’s inequality. So, in total, we have established that

$$𝔼\left[\sum_{t=1}^{T}\left(\bar{\rho }\left(A^{⋆}\right)-\bar{\rho }\left(A_{t}\right)\right)\right]=𝔼\left[\sum_{t=1}^{T}\left(\bar{\rho }\left(A^{⋆}\right)-\bar{\rho }\left({\tilde{A}}_{t}\right)\right)\right]+𝔼\left[\sum_{t=1}^{T}\left(\bar{\rho }\left({\tilde{A}}_{t}\right)-\bar{\rho }\left(A_{t}\right)\right)\right]\leq 𝔼\left[\sum_{t=1}^{T}\left(\bar{\rho }\left({\tilde{A}}_{t}\right)-\bar{\rho }\left(A_{t}\right)\right)\right]+T\sqrt{D}.$$

The remainder of the proof follows as a standard information-ratio analysis (Russo & Van Roy, 2016), only now with the provision of Lemma 2. Namely, we have

$$\begin{array}{l} 𝔼\left[\sum_{t=1}^{T}\left(\bar{\rho }\left({\tilde{A}}_{t}\right)-\bar{\rho }\left(A_{t}\right)\right)\right]=𝔼\left[\sum_{t=1}^{T}{𝔼}_{t}\left[\bar{\rho }\left({\tilde{A}}_{t}\right)-\bar{\rho }\left(A_{t}\right)\right]\right]\\ \;=𝔼\left[\sum_{t=1}^{T}\sqrt{\Gamma_{t}{𝕀}_{t}({\tilde{A}}_{t};\left(A_{t},O_{t+1}\right))}\right]\\ \;\leq \sqrt{\bar{\Gamma }}𝔼\left[\sum_{t=1}^{T}\sqrt{{𝕀}_{t}({\tilde{A}}_{t};\left(A_{t},O_{t+1}\right))}\right]\\ \;\leq \sqrt{\bar{\Gamma }T𝔼\left[\sum_{t=1}^{T}{𝕀}_{t}\left({\tilde{A}}_{t};\left(A_{t},O_{t+1}\right)\right)\right]}\\ \;\leq \sqrt{\bar{\Gamma }T{𝓡}_{1}\left(D\right)}, \end{array}$$

where the first inequality follows from our uniform upper bound to the information ratios, the second inequality is the Cauchy-Scwharz inequality, and the final inequality is due to Lemma 2. Putting everything together, we have established that

$$𝔼\left[\sum_{t=1}^{T}\left(\bar{\rho }\left(A^{⋆}\right)-\bar{\rho }\left(A_{t}\right)\right)\right]\leq \sqrt{\bar{\Gamma }T{𝓡}_{1}\left(D\right)}+T\sqrt{D}.$$

Theorem 1 then follows by Proposition 3 of Russo and Van Roy (2016), which establishes that $\bar{\Gamma }=\frac{1}{2}|𝒜|$ for a multi-armed bandit problem with rewards bounded in the unit interval and a finite action space.

## References

[bib1] Abachi, R., Ghavamzadeh, M., & Farahmand, A. (2020). Policy-aware model learning for policy gradient methods. ArXiv. 10.48550/arXiv.2003.00030

[bib2] Abbasi-Yadkori, Y., & Szepesvari, C. (2014). Bayesian optimal control of smoothly parameterized systems: The lazy posterior sampling algorithm. ArXiv. 10.48550/arXiv.1406.3926

[bib3] Abdolrahmani, M., Lyamzin, D. R., Aoki, R., & Benucci, A. (2019). Cognitive modulation of interacting corollary discharges in the visual cortex. BioRxiv. 10.1101/615229

[bib4] Abel, D., Arumugam, D., Asadi, K., Jinnai, Y., Littman, M. L., & Wong, L. L. S. (2019). State abstraction as compression in apprenticeship learning. Proceedings of the AAAI Conference on Artificial Intelligence, 33, 3134–3142. 10.1609/aaai.v33i01.33013134

[bib5] Abel, D., Jinnai, Y., Guo, S. Y., Konidaris, G., & Littman, M. (2018). Policy and value transfer in lifelong reinforcement learning. In Proceedings of the 35th international conference on machine learning (pp. 20–29). PMLR.

[bib6] Agrawal, S., & Goyal, N. (2012). Analysis of Thompson sampling for the multi-armed bandit problem. In Proceedings of the 25th annual conference on learning theory (pp. 39.1–39.26). PMLR.

[bib7] Agrawal, S., & Goyal, N. (2013). Further optimal regret bounds for Thompson sampling. In Proceedings of the sixteenth international conference on artificial intelligence and statistics (pp. 99–107). PMLR.

[bib8] Agrawal, S., & Jia, R. (2017). Optimistic posterior sampling for reinforcement learning: Worst-case regret bounds. In Advances in neural information processing systems (pp. 1184–1194).

[bib9] Amir, N., Suliman-Lavie, R., Tal, M., Shifman, S., Tishby, N., & Nelken, I. (2020). Value-complexity tradeoff explains mouse navigational learning. PLoS Computational Biology, 16(12), e1008497. 10.1371/journal.pcbi.1008497, 33306669 PMC7758052

[bib10] Anderson, J. R. (1990). The adaptive character of thought. Lawrence Erlbaum Associates, Inc. 10.4324/9780203771730

[bib11] Arimoto, S. (1972). An algorithm for computing the capacity of arbitrary discrete memoryless channels. IEEE Transactions on Information Theory, 18(1), 14–20. 10.1109/TIT.1972.1054753

[bib12] Arumugam, D., & Van Roy, B. (2021a). Deciding what to learn: A rate-distortion approach. In Proceedings of the 38th international conference on machine learning (pp. 373–382). PMLR.

[bib13] Arumugam, D., & Van Roy, B. (2021b). The value of information when deciding what to learn. In Advances in neural information processing systems (Vol. 34, pp. 9816–9827).

[bib14] Arumugam, D., & Van Roy, B. (2022). Deciding what to model: Value-equivalent sampling for reinforcement learning. In Advances in neural information processing systems (Vol. 35, pp. 9024–9044).

[bib15] Asadi, K., & Littman, M. L. (2017). An alternative softmax operator for reinforcement learning. In Proceedings of the 34th international conference on machine learning (pp. 243–252). PMLR.

[bib16] Asadi, K., Misra, D., & Littman, M. (2018). Lipschitz continuity in model-based reinforcement learning. In Proceedings of the 35th international conference on machine learning (pp. 264–273). PMLR.

[bib17] Auer, P. (2002). Using confidence bounds for exploitation-exploration trade-offs. Journal of Machine Learning Research, 3, 397–422.

[bib18] Auer, P., Cesa-Bianchi, N., & Fischer, P. (2002). Finite-time analysis of the multiarmed bandit problem. Machine Learning, 47, 235–256. 10.1023/A:1013689704352

[bib19] Auer, P., Jaksch, T., & Ortner, R. (2009). Near-optimal regret bounds for reinforcement learning. In Advances in neural information processing systems (pp. 89–96).

[bib20] Ayoub, A., Jia, Z., Szepesvari, C., Wang, M., & Yang, L. (2020). Model-based reinforcement learning with value-targeted regression. In Proceedings of the 37th international conference on machine learning (pp. 463–474). PMLR.

[bib21] Azar, M. G., Osband, I., & Munos, R. (2017). Minimax regret bounds for reinforcement learning. In Proceedings of the 34th international conference on machine learning (pp. 263–272). PMLR.

[bib22] Baker, C. L., Saxe, R., & Tenenbaum, J. B. (2009). Action understanding as inverse planning. Cognition, 113(3), 329–349. 10.1016/j.cognition.2009.07.005, 19729154

[bib23] Bari, B. A., & Gershman, S. J. (2022). Undermatching is a consequence of policy compression. BioRxiv. 10.1101/2022.05.25.493472 PMC9864556 36639891

[bib24] Bartlett, P. L., & Tewari, A. (2009). REGAL: A regularization based algorithm for reinforcement learning in weakly communicating MDPs. In Proceedings of the twenty-fifth conference on uncertainty in artificial intelligence (pp. 35–42). AUAI Press.

[bib25] Battaglia, P. W., Hamrick, J. B., & Tenenbaum, J. B. (2013). Simulation as an engine of physical scene understanding. Proceedings of the National Academy of Sciences, 110(45), 18327–18332. 10.1073/pnas.1306572110, 24145417 PMC3831455

[bib26] Bellemare, M. G., Ostrovski, G., Guez, A., Thomas, P. S., & Munos, R. (2016). Increasing the action gap: New operators for reinforcement learning. In Proceedings of the AAAI conference on artificial intelligence (Vol. 30, pp. 1476–1483).

[bib27] Bellman, R. (1957). A Markovian decision process. Journal of Mathematics and Mechanics, 6(5), 679–684. 10.1512/iumj.1957.6.56038

[bib28] Bellman, R., & Kalaba, R. (1959). On adaptive control processes. IRE Transactions on Automatic Control, 4(2), 1–9. 10.1109/TAC.1959.1104847

[bib29] Berger, T. (1971). Rate distortion theory: A mathematical basis for data compression. Prentice-Hall.

[bib30] Bertsekas, D. P. (1995). Dynamic programming and optimal control. Athena Scientific.

[bib31] Bhui, R., Lai, L., & Gershman, S. J. (2021). Resource-rational decision making. Current Opinion in Behavioral Sciences, 41, 15–21. 10.1016/j.cobeha.2021.02.015

[bib32] Binz, M., & Schulz, E. (2022). Modeling human exploration through resource-rational reinforcement learning. In Advances in neural information processing systems (pp. 31755–31768).

[bib33] Blahut, R. (1972). Computation of channel capacity and rate-distortion functions. IEEE Transactions on Information Theory, 18(4), 460–473. 10.1109/TIT.1972.1054855

[bib34] Botvinick, M., Weinstein, A., Solway, A., & Barto, A. (2015). Reinforcement learning, efficient coding, and the statistics of natural tasks. Current Opinion in Behavioral Sciences, 5, 71–77. 10.1016/j.cobeha.2015.08.009

[bib35] Boyd, S. P., & Vandenberghe, L. (2004). Convex optimization. Cambridge University Press. 10.1017/CBO9780511804441

[bib36] Brafman, R. I., & Tennenholtz, M. (2002). R-MAX - A general polynomial time algorithm for near-optimal reinforcement learning. Journal of Machine Learning Research, 3, 213–231.

[bib37] Brown, V. M., Hallquist, M. N., Frank, M. J., & Dombrovski, A. Y. (2022). Humans adaptively resolve the explore-exploit dilemma under cognitive constraints: Evidence from a multi-armed bandit task. Cognition, 229, 105233. 10.1016/j.cognition.2022.105233, 35917612 PMC9530017

[bib38] Brunskill, E., & Li, L. (2013). Sample complexity of multi-task reinforcement learning. In Proceedings of the twenty-ninth conference on uncertainty in artificial intelligence (pp. 122–131). AUAI Press.

[bib39] Brunskill, E., & Li, L. (2015). The online coupon-collector problem and its application to lifelong reinforcement learning. ArXiv. 10.48550/arXiv.1506.03379

[bib40] Bubeck, S., & Cesa-Bianchi, N. (2012). Regret analysis of stochastic and nonstochastic multi-armed bandit problems. Foundations and Trends in Machine Learning, 5(1), 1–122. 10.1561/2200000024

[bib41] Bubeck, S., & Liu, C.-Y. (2013). Prior-free and prior-dependent regret bounds for Thompson sampling. In Advances in neural information processing systems (Vol. 26, pp. 638–646).

[bib42] Callaway, F., van Opheusden, B., Gul, S., Das, P., Krueger, P. M., Griffiths, T. L., & Lieder, F. (2022). Rational use of cognitive resources in human planning. Nature Human Behaviour, 6(8), 1112–1125. 10.1038/s41562-022-01332-8, 35484209

[bib43] Cesa-Bianchi, N., & Fischer, P. (1998). Finite-time regret bounds for the multiarmed bandit problem. In Proceedings of the fifteenth international conference on machine learning (pp. 100–108). Morgan Kaufmann Publishers Inc.

[bib44] Chapelle, O., & Li, L. (2011). An empirical evaluation of Thompson sampling. In Advances in neural information processing systems (pp. 2249–2257).

[bib45] Chen, Y., Dong, P., Bai, Q., Dimakopoulou, M., Xu, W., & Zhou, Z. (2022). Society of agents: Regret bounds of concurrent Thompson sampling. In A. H. Oh, A. Agarwal, D. Belgrave, & K. Cho (Eds.), Advances in neural information processing systems (Vol. 35, pp. 7587–7598).

[bib46] Chiang, M., & Boyd, S. (2004). Geometric programming duals of channel capacity and rate distortion. IEEE Transactions on Information Theory, 50(2), 245–258. 10.1109/TIT.2003.822581

[bib47] Collins, A. G. E., & Frank, M. J. (2013). Cognitive control over learning: Creating, clustering, and generalizing task-set structure. Psychological Review, 120(1), 190–229. 10.1037/a0030852, 23356780 PMC3974273

[bib48] Cook, C., Goodman, N. D., & Schulz, L. E. (2011). Where science starts: Spontaneous experiments in preschoolers’ exploratory play. Cognition, 120(3), 341–349. 10.1016/j.cognition.2011.03.003, 21561605

[bib49] Cover, T. M., & Thomas, J. A. (2012). Elements of information theory. John Wiley & Sons. 10.1002/047174882X

[bib50] Csiszár, I. (1974a). On an extremum problem of information theory. Studia Scientiarum Mathematicarum Hungarica, 9, 57–71.

[bib51] Csiszár, I. (1974b). On the computation of rate-distortion functions (corresp.). IEEE Transactions on Information Theory, 20(1), 122–124. 10.1109/TIT.1974.1055146

[bib52] Cui, B., Chow, Y., & Ghavamzadeh, M. (2020). Control-aware representations for model-based reinforcement learning. ArXiv. 10.48550/arXiv.2006.13408

[bib53] Dann, C., & Brunskill, E. (2015). Sample complexity of episodic fixed-horizon reinforcement learning. In Proceedings of the 28th international conference on neural information processing systems - volume 2 (pp. 2818–2826).

[bib54] Dann, C., Lattimore, T., & Brunskill, E. (2017). Unifying PAC and regret: Uniform PAC bounds for episodic reinforcement learning. In Proceedings of the 31st international conference on neural information processing systems (pp. 5717–5727).

[bib55] Dauwels, J. (2005). Numerical computation of the capacity of continuous memoryless channels. In Proceedings of the 26th symposium on information theory in the BENELUX (pp. 221–228). Citeseer.

[bib56] Daw, N. D., Gershman, S. J., Seymour, B., Dayan, P., & Dolan, R. J. (2011). Model-based influences on humans’ choices and striatal prediction errors. Neuron, 69(6), 1204–1215. 10.1016/j.neuron.2011.02.027, 21435563 PMC3077926

[bib57] Dayan, P., & Niv, Y. (2008). Reinforcement learning: The good, the bad and the ugly. Current Opinion in Neurobiology, 18(2), 185–196. 10.1016/j.conb.2008.08.003, 18708140

[bib58] Decker, J. H., Otto, A. R., Daw, N. D., & Hartley, C. A. (2016). From creatures of habit to goal-directed learners: Tracking the developmental emergence of model-based reinforcement learning. Psychological Science, 27(6), 848–858. 10.1177/0956797616639301, 27084852 PMC4899156

[bib59] Der Kiureghian, A., & Ditlevsen, O. (2009). Aleatory or epistemic? Does it matter? Structural Safety, 31(2), 105–112. 10.1016/j.strusafe.2008.06.020

[bib60] Dimakopoulou, M., Osband, I., & Van Roy, B. (2018). Scalable coordinated exploration in concurrent reinforcement learning. In Advances in neural information processing systems (Vol. 31, pp. 4219–4227).

[bib61] Dimakopoulou, M., & Van Roy, B. (2018). Coordinated exploration in concurrent reinforcement learning. In Proceedings of the 35th international conference on machine learning (pp. 1271–1279). PMLR.

[bib62] Dong, S., Van Roy, B., & Zhou, Z. (2022). Simple agent, complex environment: Efficient reinforcement learning with agent states. Journal of Machine Learning Research, 23(1), 11627–11680.

[bib63] D’Oro, P., Metelli, A. M., Tirinzoni, A., Papini, M., & Restelli, M. (2020). Gradient-aware model-based policy search. Proceedings of the AAAI Conference on Artificial Intelligence, 34, 3801–3808. 10.1609/aaai.v34i04.5791

[bib64] Duchi, J. C. (2021). Lecture notes for statistics 311/electrical engineering 377. Stanford University.

[bib65] Duff, M. O. (2002). Optimal learning: Computational procedures for Bayes-adaptive Markov decision processes. University of Massachusetts Amherst.

[bib66] Dwaracherla, V., Lu, X., Ibrahimi, M., Osband, I., Wen, Z., & Van Roy, B. (2020). Hypermodels for exploration. ArXiv. 10.48550/arXiv.2006.07464

[bib67] Dwivedi, R., & Mackey, L. (2021). Generalized kernel thinning. ArXiv. 10.48550/arXiv.2110.01593

[bib68] Farahmand, A. (2011). Action-gap phenomenon in reinforcement learning. In Advances in neural information processing systems (Vol. 24, pp. 172–180).

[bib69] Farahmand, A. (2018). Iterative value-aware model learning. In Proceedings of the 32nd international conference on neural information processing systems (pp. 9090–9101).

[bib70] Farahmand, A., Barreto, A., & Nikovski, D. (2017). Value-aware loss function for model-based reinforcement learning. In Proceedings of the 20th international conference on artificial intelligence and statistics (pp. 1486–1494). PMLR.

[bib71] Fox, R., Pakman, A., & Tishby, N. (2016). Taming the noise in reinforcement learning via soft updates. In Proceedings of the thirty-second conference on uncertainty in artificial intelligence (pp. 202–211). AUAI Press.

[bib72] Galashov, A., Jayakumar, S. M., Hasenclever, L., Tirumala, D., Schwarz, J., Desjardins, G., Czarnecki, W. M., Teh, Y. W., Pascanu, R., & Heess, N. (2019). Information asymmetry in KL-regularized RL. ArXiv. 10.48550/arXiv.1905.01240

[bib73] Gelfand, I. M., & Yaglom, A. M. (1959). Calculation of the amount of information about a random function contained in another such function. American Mathematical Society.

[bib74] Gershman, S. J. (2018). Deconstructing the human algorithms for exploration. Cognition, 173, 34–42. 10.1016/j.cognition.2017.12.014, 29289795 PMC5801139

[bib75] Gershman, S. J. (2019). Uncertainty and exploration. Decision, 6(3), 277–286. 10.1037/dec0000101, 33768122 PMC7989061

[bib76] Gershman, S. J. (2020). Origin of perseveration in the trade-off between reward and complexity. Cognition, 204, 104394. 10.1016/j.cognition.2020.104394, 32679270

[bib77] Gershman, S. J. (2023). The rational analysis of memory. In M. Kahana & A. Wagner (Eds.), Oxford handbook of human memory. Oxford University Press.

[bib78] Gershman, S. J., Horvitz, E. J., & Tenenbaum, J. B. (2015). Computational rationality: A converging paradigm for intelligence in brains, minds, and machines. Science, 349(6245), 273–278. 10.1126/science.aac6076, 26185246

[bib79] Gershman, S. J., & Lai, L. (2020). The reward-complexity trade-off in schizophrenia. BioRxiv. 10.1101/2020.11.16.385013 PMC11104411 38773995

[bib80] Ghavamzadeh, M., Mannor, S., Pineau, J., & Tamar, A. (2015). Bayesian reinforcement learning: A survey. Foundations and Trends in Machine Learning, 8(5–6), 359–483. 10.1561/2200000049

[bib81] Gigerenzer, G., & Goldstein, D. G. (1996). Reasoning the fast and frugal way: Models of bounded rationality. Psychological Review, 103(4), 650–669. 10.1037/0033-295X.103.4.650, 8888650

[bib82] Goodman, N. D., & Frank, M. C. (2016). Pragmatic language interpretation as probabilistic inference. Trends in Cognitive Sciences, 20(11), 818–829. 10.1016/j.tics.2016.08.005, 27692852

[bib83] Gopalan, A., Mannor, S., & Mansour, Y. (2014). Thompson sampling for complex online problems. In Proceedings of the 31st international conference on machine learning (pp. 100–108). PMLR.

[bib84] Gottwald, S., & Braun, D. A. (2019). Bounded rational decision-making from elementary computations that reduce uncertainty. Entropy, 21(4), 375. 10.3390/e21040375, 33267089 PMC7514859

[bib85] Goyal, A., Bengio, Y., Botvinick, M., & Levine, S. (2020). The variational bandwidth bottleneck: Stochastic evaluation on an information budget. ArXiv. 10.48550/arXiv.2004.11935

[bib86] Goyal, A., Islam, R., Strouse, D., Ahmed, Z., Larochelle, H., Botvinick, M., Bengio, Y., & Levine, S. (2019). InfoBot: Transfer and exploration via the information bottleneck. ArXiv. 10.48550/arXiv.1901.10902

[bib87] Goyal, A., Sodhani, S., Binas, J., Peng, X. B., Levine, S., & Bengio, Y. (2020). Reinforcement learning with competitive ensembles of information-constrained primitives. ArXiv. 10.48550/arXiv.1906.10667

[bib88] Granmo, O.-C. (2010). Solving two-armed Bernoulli bandit problems using a Bayesian learning automaton. International Journal of Intelligent Computing and Cybernetics, 3(2), 207–234. 10.1108/17563781011049179

[bib89] Gray, R. M. (2011). Entropy and information theory. Springer. 10.1007/978-1-4419-7970-4

[bib90] Griffiths, T. L., Lieder, F., & Goodman, N. D. (2015). Rational use of cognitive resources: Levels of analysis between the computational and the algorithmic. Topics in Cognitive Science, 7(2), 217–229. 10.1111/tops.12142, 25898807

[bib91] Grimm, C., Barreto, A., Farquhar, G., Silver, D., & Singh, S. (2021). Proper value equivalence. In Advances in neural information processing systems (Vol. 34, pp. 7773–7786).

[bib92] Grimm, C., Barreto, A., & Singh, S. (2022). Approximate value equivalence. In Advances in neural information processing systems (Vol. 35, pp. 33029–33040).

[bib93] Grimm, C., Barreto, A., Singh, S., & Silver, D. (2020). The value equivalence principle for model-based reinforcement learning. In Advances in neural information processing systems (Vol. 33, pp. 5541–5552).

[bib94] Haarnoja, T., Tang, H., Abbeel, P., & Levine, S. (2017). Reinforcement learning with deep energy-based policies. In Proceedings of the 34th international conference on machine learning (Vol. 70, pp. 1352–1361). PMLR.

[bib95] Haarnoja, T., Zhou, A., Abbeel, P., & Levine, S. (2018). Soft actor-critic: Off-policy maximum entropy deep reinforcement learning with a stochastic actor. In Proceedings of the 35th international conference on machine learning (Vol. 80, pp. 1861–1870). PMLR.

[bib96] Hao, B., & Lattimore, T. (2022). Regret bounds for information-directed reinforcement learning. In Advances in neural information processing systems (Vol. 35, pp. 28575–28587).

[bib97] Hao, B., Lattimore, T., & Qin, C. (2022). Contextual information-directed sampling. In Proceedings of the 39th international conference on machine learning (pp. 8446–8464). PMLR.

[bib98] Harrison, M. T., & Kontoyiannis, I. (2008). Estimation of the rate–distortion function. IEEE Transactions on Information Theory, 54(8), 3757–3762. 10.1109/TIT.2008.926387

[bib99] Ho, M. K., Abel, D., Cohen, J. D., Littman, M. L., & Griffiths, T. L. (2020). The efficiency of human cognition reflects planned information processing. In Proceedings of the 34th AAAI conference on artificial intelligence (pp. 1300–1307). AAAI Press.

[bib100] Ho, M. K., Abel, D., Correa, C. G., Littman, M. L., Cohen, J. D., & Griffiths, T. L. (2022). People construct simplified mental representations to plan. Nature, 606(7912), 129–136. 10.1038/s41586-022-04743-9, 35589843

[bib101] Ho, M. K., & Griffiths, T. L. (2022). Cognitive science as a source of forward and inverse models of human decisions for robotics and control. Annual Review of Control, Robotics, and Autonomous Systems, 5, 33–53. 10.1146/annurev-control-042920-015547

[bib102] Icard, T., & Goodman, N. D. (2015). A resource-rational approach to the causal frame problem. In Proceedings from the 37th annual meeting of the Cognitive Science Society. Cognitive Science Society

[bib103] Isele, D., Rostami, M., & Eaton, E. (2016). Using task features for zero-shot knowledge transfer in lifelong learning. In Proceedings of the twenty-fifth international joint conference on artificial intelligence (Vol. 16, pp. 1620–1626). AAAI Press.

[bib104] Itti, L., & Baldi, P. (2009). Bayesian surprise attracts human attention. Vision Research, 49(10), 1295–1306. 10.1016/j.visres.2008.09.007, 18834898 PMC2782645

[bib105] Jakob, A. M. V., & Gershman, S. J. (2022). Rate-distortion theory of neural coding and its implications for working memory. BioRxiv. 10.1101/2022.02.28.482269 PMC10353860 37435811

[bib106] Jaksch, T., Ortner, R., & Auer, P. (2010). Near-optimal regret bounds for reinforcement learning. Journal of Machine Learning Research, 11(4), 1563–1600.

[bib107] Jaynes, E. T. (2003). Probability theory: The logic of science. Cambridge University Press. 10.1017/CBO9780511790423

[bib108] Jin, C., Allen-Zhu, Z., Bubeck, S., & Jordan, M. I. (2018). Is Q-learning provably efficient? In Proceedings of the 32nd international conference on neural information processing systems (pp. 4868–4878).

[bib109] Kaelbling, L. P., Littman, M. L., & Cassandra, A. R. (1998). Planning and acting in partially observable stochastic domains. Artificial Intelligence, 101(1–2), 99–134. 10.1016/S0004-3702(98)00023-X

[bib110] Kaelbling, L. P., Littman, M. L., & Moore, A. W. (1996). Reinforcement learning: A survey. Journal of Artificial Intelligence Research, 4, 237–285. 10.1613/jair.301

[bib111] Kakade, S. M. (2003). On the sample complexity of reinforcement learning [PhD thesis]. Gatsby Computational Neuroscience Unit, University College London.

[bib112] Kappen, H. J., Gómez, V., & Opper, M. (2012). Optimal control as a graphical model inference problem. Machine Learning, 87(2), 159–182. 10.1007/s10994-012-5278-7

[bib113] Kearns, M., & Singh, S. (2002). Near-optimal reinforcement learning in polynomial time. Machine Learning, 49(2–3), 209–232. 10.1023/A:1017984413808

[bib114] Klyubin, A. S., Polani, D., & Nehaniv, C. L. (2005). Empowerment: A universal agent-centric measure of control. In 2005 IEEE congress on evolutionary computation (Vol. 1, pp. 128–135). IEEE. 10.1109/CEC.2005.1554676

[bib115] Kocsis, L., & Szepesvári, C. (2006). Bandit based Monte-Carlo planning. In Machine learning: ECML 2006: 17th European Conference on Machine Learning, Berlin, Germany, September 18–22, 2006, Proceedings (pp. 282–293). Springer. 10.1007/11871842_29

[bib116] Konidaris, G., & Barto, A. (2006). Autonomous shaping: Knowledge transfer in reinforcement learning. In Proceedings of the 23rd international conference on machine learning (pp. 489–496). Association for Computing Machinery. 10.1145/1143844.1143906

[bib117] Körding, K. P., & Wolpert, D. M. (2004). Bayesian integration in sensorimotor learning. Nature, 427(6971), 244–247. 10.1038/nature02169, 14724638

[bib118] Kuleshov, V., & Precup, D. (2014). Algorithms for multi-armed bandit problems. ArXiv. 10.48550/arXiv.1402.6028

[bib119] Lai, L., & Gershman, S. J. (2021). Policy compression: An information bottleneck in action selection. In Psychology of learning and motivation (Vol. 74, pp. 195–232). Elsevier. 10.1016/bs.plm.2021.02.004

[bib120] Lai, T. L., & Robbins, H. (1985). Asymptotically efficient adaptive allocation rules. Advances in Applied Mathematics, 6(1), 4–22. 10.1016/0196-8858(85)90002-8

[bib121] Lake, B. M., Ullman, T. D., Tenenbaum, J. B., & Gershman, S. J. (2017). Building machines that learn and think like people. Behavioral and Brain Sciences, 40, e253. 10.1017/S0140525X16001837, 27881212

[bib122] Lattimore, T., & Gyorgy, A. (2021). Mirror descent and the information ratio. In Proceedings of thirty fourth conference on learning theory (pp. 2965–2992). PMLR.

[bib123] Lattimore, T., & Szepesvári, C. (2019). An information-theoretic approach to minimax regret in partial monitoring. In Proceedings of the thirty-second conference on learning theory (pp. 2111–2139). PMLR.

[bib124] Lattimore, T., & Szepesvári, C. (2020). Bandit algorithms. Cambridge University Press. 10.1017/9781108571401

[bib125] Lazaric, A., & Restelli, M. (2011). Transfer from Multiple MDPs. In Advances in neural information processing systems (Vol. 24, pp. 1746–1754).

[bib126] Lerch, R. A., & Sims, C. R. (2018). Policy generalization in capacity-limited reinforcement learning. OpenReview. https://openreview.net/forum?id=ByxAOoR5K7

[bib127] Lerch, R. A., & Sims, C. R. (2019). Rate-distortion theory and computationally rational reinforcement learning. In Proceedings of reinforcement learning and decision making (RLDM).

[bib128] Levine, S. (2018). Reinforcement learning and control as probabilistic inference: Tutorial and review. ArXiv. 10.48550/arXiv.1805.00909

[bib129] Lewis, R. L., Howes, A., & Singh, S. (2014). Computational rationality: Linking mechanism and behavior through bounded utility maximization. Topics in Cognitive Science, 6(2), 279–311. 10.1111/tops.12086, 24648415

[bib130] Lieder, F., & Griffiths, T. L. (2020). Resource-rational analysis: Understanding human cognition as the optimal use of limited computational resources. Behavioral and Brain Sciences, 43, e1. 10.1017/S0140525X1900061X, 30714890

[bib131] Lieder, F., Plunkett, D., Hamrick, J. B., Russell, S. J., Hay, N., & Griffiths, T. (2014). Algorithm selection by rational metareasoning as a model of human strategy selection. In Advances in neural information processing systems (pp. 2870–2878).

[bib132] Littman, M. L. (1996). Algorithms for sequential decision-making [PhD thesis]. Brown University.

[bib133] Littman, M. L. (2015). Reinforcement learning improves behaviour from evaluative feedback. Nature, 521(7553), 445–451. 10.1038/nature14540, 26017443

[bib134] Lu, X., & Van Roy, B. (2019). Information-theoretic confidence bounds for reinforcement learning. In Advances in neural information processing systems (Vol. 32, pp. 2461–2470).

[bib135] Lu, X., Van Roy, B., Dwaracherla, V., Ibrahimi, M., Osband, I., & Wen, Z. (2023). Reinforcement learning, bit by bit. Foundations and Trends in Machine Learning, 16(6), 733–865. 10.1561/2200000097

[bib136] Ma, W. J. (2012). Organizing probabilistic models of perception. Trends in Cognitive Sciences, 16(10), 511–518. 10.1016/j.tics.2012.08.010, 22981359

[bib137] Ma, W. J. (2019). Bayesian decision models: A primer. Neuron, 104(1), 164–175. 10.1016/j.neuron.2019.09.037, 31600512

[bib138] Marr, D. (1982). Vision: A computational investigation into the human representation and processing of visual information. W. H. Freeman and Company.

[bib139] Mikhael, J. G., Lai, L., & Gershman, S. J. (2021). Rational inattention and tonic dopamine. PLoS Computational Biology, 17(3), e1008659. 10.1371/journal.pcbi.1008659, 33760806 PMC7990190

[bib140] Nair, S., Savarese, S., & Finn, C. (2020). Goal-aware prediction: Learning to model what matters. In Proceedings of the 37th international conference on machine learning (pp. 7207–7219). PMLR.

[bib141] Newell, A., Shaw, J. C., & Simon, H. A. (1958). Elements of a theory of human problem solving. Psychological Review, 65(3), 151–166. 10.1037/h0048495

[bib142] Newell, A., & Simon, H. A. (1972). Human problem solving (Vol. 104). Prentice Hall.

[bib143] Nikishin, E., Abachi, R., Agarwal, R., & Bacon, P.-L. (2022). Control-oriented model-based reinforcement learning with implicit differentiation. Proceedings of the AAAI Conference on Artificial Intelligence, 36(7), 7886–7894. 10.1609/aaai.v36i7.20758

[bib144] O’Donoghue, B., Osband, I., & Ionescu, C. (2020). Making sense of reinforcement learning and probabilistic inference. ArXiv. 10.48550/arXiv.2001.00805

[bib145] Oh, J., Singh, S., & Lee, H. (2017). Value prediction network. In Proceedings of the 31st international conference on neural information processing systems (pp. 6118–6128).

[bib146] Ortega, P. A., & Braun, D. A. (2011). Information, utility and bounded rationality. In Artificial general intelligence: 4th International Conference, AGI 2011, Mountain View, CA, USA, August 3–6, 2011, Proceedings (pp. 269–274). Springer. 10.1007/978-3-642-22887-2_28

[bib147] Ortega, P. A., & Braun, D. A. (2013). Thermodynamics as a theory of decision-making with information-processing costs. Proceedings of the Royal Society A: Mathematical, Physical and Engineering Sciences, 469(2153), 20120683. 10.1098/rspa.2012.0683

[bib148] Osband, I., Blundell, C., Pritzel, A., & Van Roy, B. (2016). Deep exploration via Bootstrapped DQN. In Advances in neural information processing systems (pp. 4026–4034).

[bib149] Osband, I., Russo, D., & Van Roy, B. (2013). (More) efficient reinforcement learning via posterior sampling. In Advances in neural information processing systems (Vol. 26, pp. 3003–3011).

[bib150] Osband, I., & Van Roy, B. (2014). Model-based reinforcement learning and the Eluder dimension. In Advances in neural information processing systems (Vol. 27, pp. 1466–1474).

[bib151] Osband, I., & Van Roy, B. (2017). Why is posterior sampling better than optimism for reinforcement learning? In Proceedings of the 34th international conference on machine learning (pp. 2701–2710). PMLR.

[bib152] Osband, I., Van Roy, B., Russo, D. J., & Wen, Z. (2019). Deep exploration via randomized value functions. Journal of Machine Learning Research, 20(124), 1–62.

[bib153] Osband, I., Van Roy, B., & Wen, Z. (2016). Generalization and exploration via randomized value functions. In Proceedings of the 33rd international conference on machine learning (pp. 2377–2386). PMLR.

[bib154] Palaiyanur, H., & Sahai, A. (2008). On the uniform continuity of the rate-distortion function. In 2008 IEEE international symposium on information theory (pp. 857–861). IEEE. 10.1109/ISIT.2008.4595108

[bib155] Parush, N., Tishby, N., & Bergman, H. (2011). Dopaminergic balance between reward maximization and policy complexity. Frontiers in Systems Neuroscience, 5, 22. 10.3389/fnsys.2011.00022, 21603228 PMC3093748

[bib156] Peng, L. (2005). Learning with information capacity constraints. Journal of Financial and Quantitative Analysis, 40(2), 307–329. 10.1017/S0022109000002325

[bib157] Perez, A. (1959). Information theory with an abstract alphabet (generalized forms of McMillan’s limit theorem for the case of discrete and continuous times). Theory of Probability & Its Applications, 4(1), 99–102. 10.1137/1104007

[bib158] Polani, D. (2009). Information: Currency of life? HFSP Journal, 3(5), 307–316. 10.2976/1.3171566, 20357888 PMC2801531

[bib159] Polani, D. (2011). An informational perspective on how the embodiment can relieve cognitive burden. In 2011 IEEE symposium on artificial life (ALIFE) (pp. 78–85). IEEE. 10.1109/ALIFE.2011.5954666

[bib160] Polyanskiy, Y., & Wu, Y. (2024). Information theory: From coding to learning. Cambridge University Press.

[bib161] Powell, W. B., & Ryzhov, I. O. (2012). Optimal learning (Vol. 841). John Wiley & Sons. 10.1002/9781118309858

[bib162] Prystawski, B., Mohnert, F., Tošić, M., & Lieder, F. (2022). Resource-rational models of human goal pursuit. Topics in Cognitive Science, 14(3), 528–549. 10.1111/tops.12562, 34435728

[bib163] Puterman, M. L. (1994). Markov decision processes: Discrete stochastic dynamic programming. John Wiley & Sons, Inc. 10.1002/9780470316887

[bib164] Radulescu, A., Niv, Y., & Ballard, I. (2019). Holistic reinforcement learning: The role of structure and attention. Trends in Cognitive Sciences, 23(4), 278–292. 10.1016/j.tics.2019.01.010, 30824227 PMC6472955

[bib165] Rubin, J., Shamir, O., & Tishby, N. (2012). Trading value and information in MDPs. In Decision making with imperfect decision makers (pp. 57–74). Springer. 10.1007/978-3-642-24647-0_3

[bib166] Russo, D., & Van Roy, B. (2014). Learning to optimize via information-directed sampling. In Advances in neural information processing systems (Vol. 27, pp. 1583–1591).

[bib167] Russo, D., & Van Roy, B. (2016). An information-theoretic analysis of Thompson sampling. Journal of Machine Learning Research, 17(1), 2442–2471.

[bib168] Russo, D., & Van Roy, B. (2018a). Learning to optimize via information-directed sampling. Operations Research, 66(1), 230–252. 10.1287/opre.2017.1663

[bib169] Russo, D., & Van Roy, B. (2018b). Satisficing in time-sensitive bandit learning. ArXiv. 10.48550/arXiv.1803.02855

[bib170] Russo, D., & Van Roy, B. (2022). Satisficing in time-sensitive bandit learning. Mathematics of Operations Research, 47(4), 2815–2839. 10.1287/moor.2021.1229

[bib171] Russo, D. J., Van Roy, B., Kazerouni, A., Osband, I., & Wen, Z. (2018). A tutorial on Thompson sampling. Foundations and Trends in Machine Learning, 11(1), 1–96. 10.1561/2200000070

[bib172] Ryzhov, I. O., Powell, W. B., & Frazier, P. I. (2012). The knowledge gradient algorithm for a general class of online learning problems. Operations Research, 60(1), 180–195. 10.1287/opre.1110.0999

[bib173] Schrittwieser, J., Antonoglou, I., Hubert, T., Simonyan, K., Sifre, L., Schmitt, S., Guez, A., Lockhart, E., Hassabis, D., Graepel, T., Lillicrap, T., & Silver, D. (2020). Mastering Atari, Go, chess and shogi by planning with a learned model. Nature, 588(7839), 604–609. 10.1038/s41586-020-03051-4, 33361790

[bib174] Schulz, E., & Gershman, S. J. (2019). The algorithmic architecture of exploration in the human brain. Current Opinion in Neurobiology, 55, 7–14. 10.1016/j.conb.2018.11.003, 30529148

[bib175] Scott, S. L. (2010). A modern Bayesian look at the multi-armed bandit. Applied Stochastic Models in Business and Industry, 26(6), 639–658. 10.1002/asmb.874

[bib176] Shafieepoorfard, E., Raginsky, M., & Meyn, S. P. (2016). Rationally inattentive control of Markov processes. SIAM Journal on Control and Optimization, 54(2), 987–1016. 10.1137/15M1008476

[bib177] Shannon, C. E. (1948). A mathematical theory of communication. The Bell System Technical Journal, 27(3), 379–423. 10.1002/j.1538-7305.1948.tb01338.x

[bib178] Shannon, C. E. (1959). Coding theorems for a discrete source with a fidelity criterion. In Institute of radio engineers, international convention record (Vol. 4, pp. 142–163). Wiley-IEEE Press. 10.1109/9780470544242.ch21

[bib179] Shugan, S. M. (1980). The cost of thinking. Journal of Consumer Research, 7(2), 99–111. 10.1086/208799

[bib180] Silver, D., Hasselt, H., Hessel, M., Schaul, T., Guez, A., Harley, T., Dulac-Arnold, G., Reichert, D., Rabinowitz, N., Barreto, A., & Degris, T. (2017). The predictron: End-to-end learning and planning. In Proceedings of the 34th international conference on machine learning (pp. 3191–3199). PMLR.

[bib181] Simon, H. A. (1955). A behavioral model of rational choice. Quarterly Journal of Economics, 69(1), 99–118. 10.2307/1884852

[bib182] Simon, H. A. (1956). Rational choice and the structure of the environment. Psychological Review, 63(2), 129–138. 10.1037/h0042769, 13310708

[bib183] Simon, H. A. (1982). Models of bounded rationality: Economic analysis and public policy. MIT Press.

[bib184] Sims, C. A. (2003). Implications of rational inattention. Journal of Monetary Economics, 50(3), 665–690. 10.1016/S0304-3932(03)00029-1

[bib185] Sims, C. R. (2016). Rate-distortion theory and human perception. Cognition, 152, 181–198. 10.1016/j.cognition.2016.03.020, 27107330

[bib186] Sims, C. R. (2018). Efficient coding explains the universal law of generalization in human perception. Science, 360(6389), 652–656. 10.1126/science.aaq1118, 29748284

[bib187] Still, S., & Precup, D. (2012). An information-theoretic approach to curiosity-driven reinforcement learning. Theory in Biosciences, 131(3), 139–148. 10.1007/s12064-011-0142-z, 22791268

[bib188] Strehl, A. L., Li, L., & Littman, M. L. (2009). Reinforcement learning in finite MDPs: PAC analysis. Journal of Machine Learning Research, 10, 2413–2444.

[bib189] Strens, M. J. (2000). A Bayesian framework for reinforcement learning. In Proceedings of the seventeenth international conference on machine learning (pp. 943–950). Morgan Kaufmann Publishers Inc.

[bib190] Stringer, C., Michaelos, M., Tsyboulski, D., Lindo, S. E., & Pachitariu, M. (2021). High-precision coding in visual cortex. Cell, 184(10), 2767–2778. 10.1016/j.cell.2021.03.042, 33857423

[bib191] Sutton, R. S. (1991). Dyna, an integrated architecture for learning, planning, and reacting. ACM Sigart Bulletin, 2(4), 160–163. 10.1145/122344.122377

[bib192] Sutton, R. S., & Barto, A. G. (1998). Reinforcement learning: An introduction. MIT Press.

[bib193] Tenenbaum, J. B., Kemp, C., Griffiths, T. L., & Goodman, N. D. (2011). How to grow a mind: Statistics, structure, and abstraction. Science, 331(6022), 1279–1285. 10.1126/science.1192788, 21393536

[bib194] Thompson, W. R. (1933). On the likelihood that one unknown probability exceeds another in view of the evidence of two samples. Biometrika, 25(3–4), 285–294. 10.1093/biomet/25.3-4.285

[bib195] Thrun, S., & Schwartz, A. (1994). Finding structure in reinforcement learning. In Advances in neural information processing systems (Vol. 7, pp. 385–392).

[bib196] Tiomkin, S., & Tishby, N. (2017). A unified Bellman equation for causal information and value in Markov decision processes. ArXiv. 10.48550/arXiv.1703.01585

[bib197] Tirumala, D., Noh, H., Galashov, A., Hasenclever, L., Ahuja, A., Wayne, G., Pascanu, R., Teh, Y. W., & Heess, N. (2019). Exploiting hierarchy for learning and transfer in KL-regularized RL. ArXiv. 10.48550/arXiv.1903.07438

[bib198] Tishby, N., & Polani, D. (2011). Information theory of decisions and actions. In Perception-action cycle: Models, architectures, and hardware (pp. 601–636). Springer. 10.1007/978-1-4419-1452-1_19

[bib199] Todorov, E. (2007). Linearly-solvable Markov decision problems. In Advances in neural information processing systems (pp. 1369–1376). MIT Press. 10.7551/mitpress/7503.003.0176

[bib200] Toussaint, M. (2009). Robot trajectory optimization using approximate inference. In Proceedings of the 26th annual international conference on machine learning (pp. 1049–1056). 10.1145/1553374.1553508

[bib201] Vermorel, J., & Mohri, M. (2005). Multi-armed bandit algorithms and empirical evaluation. In Machine learning: ECML 2005: 16th European conference on machine learning, Porto, Portugal, October 3–7, 2005 (pp. 437–448). Springer. 10.1007/11564096_42

[bib202] Voelcker, C. A., Liao, V., Garg, A., & Farahmand, A. (2022). Value gradient weighted model-based reinforcement learning. ArXiv. 10.48550/arXiv.2204.01464

[bib203] von Neumann, J., & Morgenstern, O. (1944). Theory of games and economic behavior. Princeton University Press.

[bib204] Vul, E., Goodman, N., Griffiths, T. L., & Tenenbaum, J. B. (2014). One and done? Optimal decisions from very few samples. Cognitive Science, 38(4), 599–637. 10.1111/cogs.12101, 24467492

[bib205] Vulkan, N. (2000). An economist’s perspective on probability matching. Journal of Economic Surveys, 14(1), 101–118. 10.1111/1467-6419.00106

[bib206] Wilson, A., Fern, A., Ray, S., & Tadepalli, P. (2007). Multi-task reinforcement learning: A hierarchical Bayesian approach. In Proceedings of the 24th international conference on machine learning (pp. 1015–1022). 10.1145/1273496.1273624

[bib207] Wilson, R. C., & Collins, A. G. (2019). Ten simple rules for the computational modeling of behavioral data. Elife, 8, e49547. 10.7554/eLife.49547, 31769410 PMC6879303

[bib208] Wilson, R. C., Geana, A., White, J. M., Ludvig, E. A., & Cohen, J. D. (2014). Humans use directed and random exploration to solve the explore-exploit dilemma. Journal of Experimental Psychology: General, 143(6), 2074–2081. 10.1037/a0038199, 25347535 PMC5635655

[bib209] Wozny, D. R., Beierholm, U. R., & Shams, L. (2010). Probability matching as a computational strategy used in perception. PLoS Computational Biology, 6(8), e1000871. 10.1371/journal.pcbi.1000871, 20700493 PMC2916852

[bib210] Yuille, A., & Kersten, D. (2006). Vision as Bayesian inference: Analysis by synthesis? Trends in Cognitive Sciences, 10(7), 301–308. 10.1016/j.tics.2006.05.002, 16784882

[bib211] Zanette, A., & Brunskill, E. (2019). Tighter problem-dependent regret bounds in reinforcement learning without domain knowledge using value function bounds. In Proceedings of the 36th international conference on machine learning (pp. 7304–7312). PMLR.

[bib212] Zaslavsky, N., Hu, J., & Levy, R. P. (2021). A rate–distortion view of human pragmatic reasoning? In Proceedings of the society for computation in linguistics 2021 (pp. 347–348). Association for Computational Linguistics.

[bib213] Zénon, A., Solopchuk, O., & Pezzulo, G. (2019). An information-theoretic perspective on the costs of cognition. Neuropsychologia, 123, 5–18. 10.1016/j.neuropsychologia.2018.09.013, 30268880

[bib214] Ziebart, B. D. (2010). Modeling purposeful adaptive behavior with the principle of maximum causal entropy [PhD thesis]. Carnegie Mellon University.

[bib215] Zimmert, J., & Lattimore, T. (2019). Connections between mirror descent, Thompson sampling and the information ratio. In Advances in neural information processing systems (pp. 11973–11982).

